# Highly Pathogenic Avian Influenza Clade 2.3.4.4b Subtype H5N8 Virus Isolated from Mandarin Duck in South Korea, 2020

**DOI:** 10.3390/v12121389

**Published:** 2020-12-04

**Authors:** Sol Jeong, Dong-Hun Lee, Jung-Hoon Kwon, Yu-Jin Kim, Sun-Hak Lee, Andrew Y. Cho, Tae-Hyeon Kim, Jung-Eun Park, Song-I Lee, Chang-Seon Song

**Affiliations:** 1 Avian Diseases Laboratory, College of Veterinary Medicine, Konkuk University, Seoul 05029, Korea; soljeong492@gmail.com (S.J.); yujinml@hanmail.net (Y.-J.K.); cnescis@naver.com (S.-H.L.); 7rewcho@gmail.com (A.Y.C.); sakye12@naver.com (T.-H.K.); 2 Department of Pathobiology and Veterinary Science, The University of Connecticut, Storrs, CT 06269, USA; dong-hun.lee@uconn.edu; 3 Laboratory of Veterinary Microbiology, College of Veterinary Medicine, Kyungpook National University, Daegu 41566, Korea; junghoon.kwon@knu.ac.kr; 4 Wildlife Disease Response Team, National Institute of Wildlife Disease Control and Prevention, Gwangju 62407, Korea; jepark87.11@gmail.com; 5 Wildlife Disease Research Team, National Institute of Wildlife Disease Control and Prevention, Gwangju 62407, Korea; firend912@naver.com

**Keywords:** highly pathogenic avian influenza virus, wild bird, surveillance, H5N8

## Abstract

In October 2020, a highly pathogenic avian influenza (HPAI) subtype H5N8 virus was identified from a fecal sample of a wild mandarin duck (*Aix galericulata*) in South Korea. We sequenced all eight genome segments of the virus, designated as A/Mandarin duck/Korea/K20-551-4/2020(H5N8), and conducted genetic characterization and comparative phylogenetic analysis to track its origin. Genome sequencing and phylogenetic analysis show that the hemagglutinin gene belongs to H5 clade 2.3.4.4 subgroup B. All genes share high levels of nucleotide identity with H5N8 HPAI viruses identified from Europe during early 2020. Enhanced active surveillance in wild and domestic birds is needed to monitor the introduction and spread of HPAI via wild birds and to inform the design of improved prevention and control strategies.

## 1. Introduction

Highly pathogenic avian influenza (HPAI) subtype H5Nx viruses have been causing substantial losses to the poultry industry and public health concerns since the detection of A/Goose/Guangdong/1/1996(H5N1) (Gs/GD) from domestic poultry in southern China. The descendent Gs/GD-lineage H5 viruses have evolved into 10 genetically independent hemagglutinin (HA) clades (0–9) and subclades [1]. Wild birds, especially wild waterfowl of *Anatidae* family, are known as a natural reservoir of avian influenza virus (AIV) [2]. Migratory wild birds have been widely suggested to contribute to long-distance transmission and reassortment of HPAI along their migration flyways while they move between wintering and breeding sites seasonally [3,4,5,6,7].

Since the first identification of HPAI H5N5 virus in China in 2008, Gs/Gd-lineage clade 2.3.4 HPAI H5Nx viruses have evolved into subclades 2.3.4.1–2.3.4.4 [8]. Particularly, the novel clade 2.3.4.4 HPAI H5Nx viruses containing multiple neuraminidase (NA) subtypes including H5N2, H5N5, H5N6, and H5N8 have been causing numerous outbreaks in wild birds and poultry in broad geographical regions including Asia, Europe, Africa, and North America since 2014 [9]. The H5 genes of clade 2.3.4.4 HPAI H5Nx viruses were phylogenetically classified into 4 subgroups (A–D) [10]. Subgroup A and B viruses have spread over a broad geographic region due to migratory wild birds [9], and subgroup C and D viruses have been circulating mainly in China and neighboring countries in Asia [9,10,11].

During May–June 2016, novel reassortant clade 2.3.4.4 subgroup B H5N8 viruses containing internal genes of Eurasian low-pathogenic avian influenza (LPAI) viruses were identified in wild birds in Uvs Lake, Russia and Qinghai Lake, China [12,13]. These viruses had undergone further reassortments with prevailing LPAI viruses in wild birds and spread to Europe, Africa, and Asia in winter 2016–17 following the migration of wild birds [14,15,16,17,18]. In addition, these viruses further spread over large geographical areas in Asia, Africa, and Europe through wild birds and poultry trade in 2017–2018 winter [19,20,21,22]. 

The clade 2.3.4.4 subgroup B H5Nx viruses caused multiple outbreaks in South Korea in winter 2016–17 and 2017–18, which were most likely introduced by wild waterfowl [18,19,20,21,22,23]. The clade 2.3.4.4 subgroup B strains responsible for the outbreaks in South Korea had not been detected in the country since April 2018, despite large-scale active surveillance targeting both wild birds and poultry. In the present study, we report isolation of a clade 2.3.4.4 subgroup B HPAI subtype H5N8 virus from a fecal sample of a mandarin duck (*Aix galericulata*) collected in October 2020 during active wild bird surveillance for AIV in South Korea. We sequenced all eight genome segments of the virus, designated as A/Mandarin duck/Korea/K20-551-4/2020(H5N8), and conducted genetic characterization and comparative phylogenetic analysis to track its origin. 

## 2. Materials and Methods 

### 2.1. Sample Collection

We collected a total of 150 fresh fecal samples in wild bird habitat near Cheongmi Stream (GPS 37°07′09.0″ N 127°24′01.0″ E) in Anseong, South Korea, for active surveillance purposes. The fecal samples were stored at 4–8 ℃ and analyzed within 12 h.

### 2.2. Virus Isolation 

Fecal samples were placed in phosphate-buffered saline (PBS) containing 400 mg/mL gentamicin and thoroughly homogenized by vortexing for 1 min. The supernatant of samples were filtered using a 0.45 µm Minisart Syringe Filter (Sartorius, Göttingen, Germany) and inoculated into 10-day-old specific-pathogen-free (SPF) embryonated chicken eggs. After 72 h of incubation at 37 ℃, the allantoic fluids were harvested and tested for hemagglutinin activity using 10% chicken red blood cells. RNA was extracted from the hemagglutinin-activity-positive allantoic fluid using the RNeasy Mini Kit (Qiagen, Hilden, Germany) according to the manufacturer’s instruction and screened for the matrix (M) gene of avian influenza virus using real-time reverse transcription-PCR (rRT-PCR) as previously described [24]. 

### 2.3. Sequencing

Reverse transcription was conducted using SuperScript IV First-Strand Synthesis System (Invitrogen, Carslbad, CA, USA) and the eight gene segments were amplified as previously described using TaKaRa Ex Taq (Takara Bio Inc., Kusatsu, Japan) according to the manufacturer’s instruction [25]. The PCR products were purified using GeneJET Gel Extraction Kit (Thermo Scientific, Waltham, MA, USA) or QIAquick PCR Purification kit (Qiagen, Hilden, Germany) and sequenced using the ABI 3730xl DNA Analyzer (Applied Biosystems, Foster City, CA, USA). The genome sequences have been deposited in GISAID with isolate ID EPI_ISL_666687.

### 2.4. Phylogenetic Analysis

The sequences of the highly pathogenic and low-pathogenic avian influenza viruses were retrieved from the GISAID Epiflu (https://platform.gisaid.org/) database and the NCBI’s Influenza Virus Resource at GenBank (https://www.ncbi.nlm.nih.gov/genomes/FLU) (acknowledgement of the research laboratories that contributed these data is provided in Supplementary Table S1) for comparative phylogenetic analysis. Complete coding regions of the retrieved sequences were aligned using MAFFT (https://mafft.cbrc.jp/alignment/software/). The maximum-likelihood (ML) phylogenetic tree for each genome segment was constructed using RAxML v8 [26] using the general time-reversible (GTR) nucleotide substitution model and discrete gamma distribution with 1000 rapid bootstrap replicates. 

### 2.5. Host Species Identification

The host species of the AIV-positive fecal sample was identified using DNA barcoding technique as previously described [27]. Briefly, host DNA was extracted using QIAamp Fast DNA Stool Mini kit (Qiagen, Hilden, Germany) according to the manufacturer’s instruction. The mitochondrial cytochrome oxidase (COI) gene was amplified using Aves-F (5′-GCATGAGCAGGAATAGTTGG-3′) and Aves-R (5′-AAGATGTAGACTTCTGGGTG-3′) primers and sequenced using the ABI 3730xl DNA Analyzer (Applied Biosystems, Foster City, CA, USA).

## 3. Results and Discussion

On 24 October in 2020, AIV virus was isolated from the fecal sample of a mandarin duck which was confirmed based on the rRT-PCR and DNA barcoding results. The isolate A/Mandarin duck/Korea/K20-551-4/2020(H5N8), hereafter referred to as 551-4/2020 virus, was identified as an HPAI virus on the basis of multiple basic amino acids at the HA proteolytic cleavage site (PLREKRRKR/G). The GISAID database BLAST searches showed that all eight gene segments of the 551-4/2020 virus shared high nucleotide identity (>99.05%) with HPAI H5N8 viruses identified from poultry farms and wild birds in Europe in early 2020 (Table 1). 

**Figure 1:**
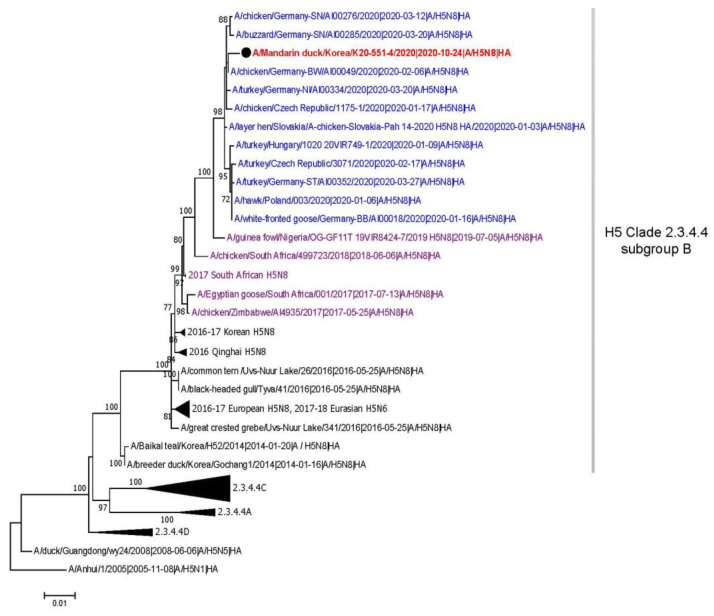
Maximum-likelihood phylogenetic tree of hemagglutinin gene. The A/Mandarin duck/Korea/K20-551-4/2020(H5N8) is indicated with black circle and colored with red. Taxa colored with blue and purple indicate H5N8 highly pathogenic avian influenza (HPAI) viruses identified in Europe during early 2020 and Africa during 2017–2019, respectively. Bootstrap values over 70% are shown next to the branches. Scale bar indicates nucleotide substitutions per site. Collapsed branches are shown in black triangles.

In the (ML) phylogenies, all the eight gene segments of 551-4/2020 virus were closely clustered together with the clade 2.3.4.4 subgroup B subtype H5N8 viruses detected from poultry and wild birds in Europe during early 2020 (Figure 1, Supplementary Figure S1). All genes of the 551-4/2020 virus were phylogenetically distinct from those of clade 2.3.4.4b H5 HPAI viruses isolated in South Korea during 2016–2018.

South Korea is located in the East Asian–Australasian flyway and serves as a wintering and stopover site for wild migratory birds [7,28,29]. Wetland near the Cheongmi Stream from where the 551-4/2020 virus was isolated is a wintering site of wild migratory *Anatidae* birds including mallard (*Anas platyrhynchos*), spot-billed duck (*Anas poecilorhyncha*), and common teal (*Anas crecca*) and also the previous outbreak site of clade 2.3.4.4 subgroup B H5N6 in winter 2017–18 [23]. During the sampling near Cheongmi Stream, we observed flocks of spot-billed ducks and mandarin ducks.

Mandarin ducks are native to the Far and Southeast of Asia including China, North and South Korea, Japan, and Eastern Russia along the East Asian–Australasian flyway. Some mandarin ducks are resident in South Korea, and others are passage migrants that occasionally overwinter in Korea and move to and from southern Japan [30]. The identifications of HPAI H5 viruses from mandarin ducks have been reported multiple times in South Korea; clade 2.3.2.1 H5N1 in 2010 [31], clade 2.3.4.4 subgroup A H5N8 in December 2014 [32], clade 2.3.4.4 subgroup C H5N6 in October 2016 [33], and clade 2.3.4.4 subgroup B H5N6 in November 2017 [23]. Previous pathogenicity studies of clade 2.3.4.4 H5Nx viruses in mandarin ducks demonstrated that mandarin ducks can excrete virus without showing clinical signs or mortality and also transmit virus to other ducks by contact, indicating they can serve as healthy carriers of clade 2.3.4.4 H5Nx viruses [34,35,36]. Because the Mandarin duck is not a long-distance migrating species, the other species of long-distance migrants were suspected as an intermediate vector for this long-distance transmission. 

The timing and location of the 551-4/2020 virus isolation, lack of identification of related clade 2.3.4.4 subgroup B H5Nx virus in South Korea in recent two and half years, and phylogenetic analysis and BLAST search results collectively suggest that migratory wild birds might have introduced the 551-4/2020 virus into South Korea during the fall migration of wild birds. However, the detailed transmission route of this virus could not be determined because of the lack of wild bird surveillance data during spring and summer 2020. It is most likely that the subgroup B H5Nx viruses belonging to clade 2.3.4.4 affected Europe during early 2020 and were then maintained in wild birds before being introduced into South Korea in fall 2020. Previous studies showed that numerous wild waterfowl species can be infected by clade 2.3.4.4 viruses without detectable clinical signs [37,38], and this biological characteristic makes difficult to detect viruses from wild waterfowl, supporting the hypothesis that wild birds in Eurasia maintained the virus and long-distance migrants introduced the virus into South Korea.

Considering the continued emergence of novel reassortant viruses and global dissemination of the clade 2.3.4.4 HPAI H5Nx viruses, enhanced active surveillance in wild and domestic birds is required to monitor the introduction, spread, and reassortment of HPAI and to inform the design of improved prevention and control strategies.

## Figures and Tables

**Table 1:** Nucleotide sequence identities between the A/Mandarin duck/Korea/K20-551-4/2020(H5N8) virus and nearest virus homologs in the GISAID Epiflu database (as of 2 November 2020).

Gene ^1^	Accession No.	Virus	% Identity
*PB2*	EPI1691234	A/chicken/Germany-BW/AI00049/2020 (H5N8)	99.56
	EPI1669674	A/hawk/Poland/003/2020 (H5N8)	
*PB1*	EPI1721422	A/duck/Hungary/1565_20VIR749-2/2020 (H5N8)	99.34
*PA*	EPI1718616	A/buzzard/Germany-SN/AI00285/2020 (H5N8)	99.58
*HA*	EPI1691237	A/chicken/Germany-BW/AI00049/2020 (H5N8)	99.65
*NP*	EPI1721665	A/turkey/Hungary/1020_20VIR749-1/2020 (H5N8)	99.33
	EPI1691238	A/chicken/Germany-BW/AI00049/2020 (H5N8)	
	EPI1665425	A/white-fronted goose/Germany-BB/AI00018/2020 (H5N8)	
*NA*	EPI1691239	A/chicken/Germany-BW/AI00049/2020 (H5N8)	99.08
	EPI1669671	A/turkey/Poland/23/2019 (H5N8)	
*MP*	EPI1721683	A/turkey/Germany-ST/AI00352/2020 (H5N8)	99.80
	EPI1718620	A/turkey/Germany-NI/AI00334/2020	
	EPI1691240	A/chicken/Germany-BW/AI00049/2020	
	EPI1669680	A/hawk/Poland/003/2020	
	EPI1669672	A/turkey/Poland/23/2019	
	EPI1665427	A/white-fronted goose/Germany-BB/AI00018/2020	
*NS*	EPI1719046	A/turkey/Czech Republic/3071/2020 (H5N8)	99.05

^1^ *PB2, basic polymerase 2; PB1, basic polymerase 1; PA, acidic polymerase; HA, hemagglutinin; NP, nucleoprotein; NA, neuraminidase; MP, matrix protein; NS, nonstructural protein.*

## Supplementary Materials

The following are available online at https://www.mdpi.com/1999-4915/12/12/1389/s1, Figure S1: Maximum-likelihood phylogenetic trees of eight gene segments. The A/Mandarin duck/Korea/K20-551-4/2020(H5N8) is indicated with black circle. Bootstrap values over 70% are shown next to the branches. Scale bar indicates nucleotide substitutions. (A) *Polymerase basic 2* (*PB2*); (B) *polymerase basic 1* (*PB1*); (C) *polymerase acidic* (*PA*); (D) *hemagglutinin* (*HA*); (E) *nucleoprotein* (*NP*); (F) *neuraminidase* (*NA*); (G) *matrix* (*M*); (H) *nonstructural* (*NS*). Table S1: The sequences from the GISAID Epiflu Database and NCBI’s Influenza Virus Resources at GenBank, on which this research is based.
