# Supplementary material for: Highly Pathogenic Avian Influenza Clade 2.3.4.4b Subtype H5N8 Virus Isolated from Mandarin Duck in South Korea, 2020
Source: Viruses. 2020 Dec 4;12(12):1389. doi: 10.3390/v12121389 (PMC7761861; doi:10.3390/v12121389)
Supplement: Supplementary file 1 [file viruses-12-01389-s001.zip › supplementary/figures.pdf]

Supplementary Figure S1. Maximum-likelihood phylogenetic trees of the PB2, PB1, PA, HA, NP, NA, MP, and NS genes. Taxa was colored according to the isolation location (red, Mongolia; blue, Xinjiang, China). The subgroup B H5N8 isolates that are closely related to the 551-4/2020 virus are colored according to the isolation location. Bootstrap values over 70% are shown next to the branches Each cluster is labeled according to the genotype classification by King et al. [1] and Baek and Lee et al. [2]. A, polymerase basic 2 (PB2); B, polymerase basic 1 (PB1); C, polymerase acidic (PA); D, haemagglutinin (HA); E, nucleoprotein (NP); F, neuraminidase (NA); G, matrix (M); H, nonstructual (NS).

References

1. King, J.; Harder, T.; Conraths, F. J.; Beer, M.; Pohlmann, A., The genetics of highly pathogenic avian influenza viruses of subtype H5 in Germany, 2006-2020. Transbound Emerg Dis 2020.

2. Baek, Y. G.; Lee, Y. N.; Lee, D. H.; Cheon, S. H.; Kye, S. J.; Park, Y. R.; Si, Y. J.; Lee, M. H.; Lee, Y. J., A novel reassortant clade 2.3.4.4 highly pathogenic avian influenza H5N6 virus identified in South Korea in 2018. Infect Genet Evol 2020, 78, 104056.

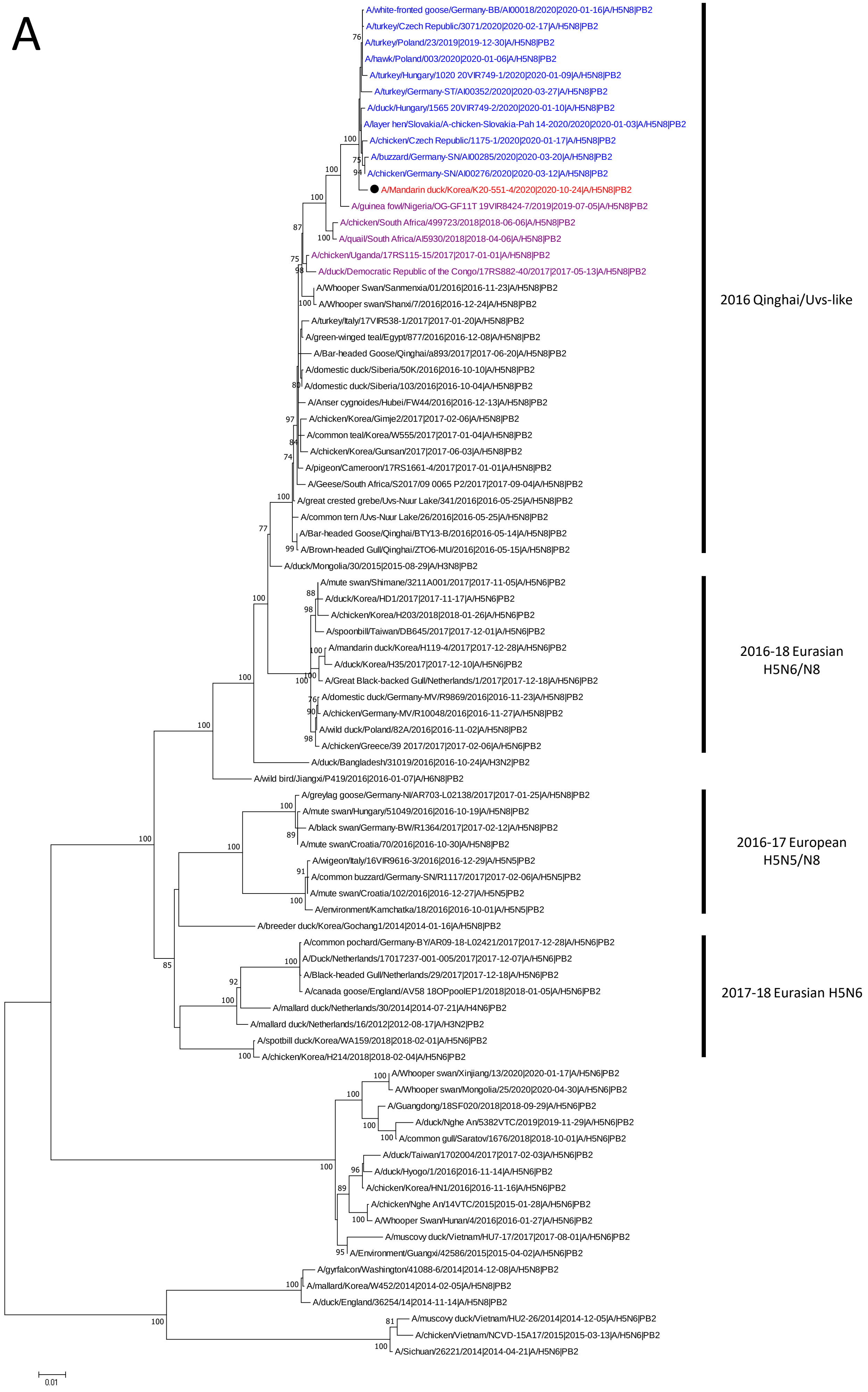
A
A/white-fronted goose/Germany-BB/AI00018/2020|2020-01-16|A/H5N8|PB2
A/turkey/Czech Republic/3071/2020|2020-02-17|A/H5N8|PB2
A/turkey/Poland/23/2019|2019-12-30|A/H5N8|PB2
A/hawk/Poland/003/2020|2020-01-06|A/H5N8|PB2
A/turkey/Hungary/1020 20VIR749-1/2020|2020-01-09|A/H5N8|PB2
A/turkey/Germany-ST/AI00352/2020|2020-03-27|A/H5N8|PB2
A/duck/Hungary/1565 20VIR749-2/2020|2020-01-10|A/H5N8|PB2
A/layer hen/Slovakia/A-chicken-Slovakia-Pah 14-2020/2020|2020-01-03|A/H5N8|PB2
A/chicken/Czech Republic/1175-1/2020|2020-01-17|A/H5N8|PB2
A/buzzard/Germany-SN/AI00285/2020|2020-03-20|A/H5N8|PB2
A/chicken/Germany-SN/AI00276/2020|2020-03-12|A/H5N8|PB2
A/Mandarin duck/Korea/K20-551-4/2020|2020-10-24|A/H5N8|PB2
A/guinea fowl/Nigeria/OG-GF11T 19VIR8424-7/2019|2019-07-05|A/H5N8|PB2
A/chicken/South Africa/499723/2018|2018-06-06|A/H5N8|PB2
A/quail/South Africa/AI5930/2018|2018-04-06|A/H5N8|PB2
A/chicken/Uganda/17RS115-15/2017|2017-01-01|A/H5N8|PB2
A/duck/Democratic Republic of the Congo/17RS882-40/2017|2017-05-13|A/H5N8|PB2
A/Whooper Swan/Sanmenxia/01/2016|2016-11-23|A/H5N8|PB2
A/Whooper swan/Shanxi/7/2016|2016-12-24|A/H5N8|PB2
A/turkey/Italy/17VIR538-1/2017|2017-01-20|A/H5N8|PB2
A/green-winged teal/Egypt/877/2016|2016-12-08|A/H5N8|PB2
A/Bar-headed Goose/Qinghai/a893/2017|2017-06-20|A/H5N8|PB2
A/domestic duck/Siberia/50K/2016|2016-10-10|A/H5N8|PB2
A/domestic duck/Siberia/103/2016|2016-10-04|A/H5N8|PB2
A/Anser cygnoides/Hubei/FW44/2016|2016-12-13|A/H5N8|PB2
A/chicken/Korea/Gimje2/2017|2017-02-06|A/H5N8|PB2
A/common teal/Korea/W555/2017|2017-01-04|A/H5N8|PB2
A/chicken/Korea/Gunsan/2017|2017-06-03|A/H5N8|PB2
A/pigeon/Cameroon/17RS1661-4/2017|2017-01-01|A/H5N8|PB2
A/Geese/South Africa/S2017/09 0065 P2/2017|2017-09-04|A/H5N8|PB2
A/great crested grebe/Uvs-Nuur Lake/341/2016|2016-05-25|A/H5N8|PB2
A/common tern /Uvs-Nuur Lake/26/2016|2016-05-25|A/H5N8|PB2
A/Bar-headed Goose/Qinghai/BTY13-B/2016|2016-05-14|A/H5N8|PB2
A/Brown-headed Gull/Qinghai/ZTO6-MU/2016|2016-05-15|A/H5N8|PB2
A/duck/Mongolia/30/2015|2015-08-29|A/H3N8|PB2
A/mute swan/Shimane/3211A001/2017|2017-11-05|A/H5N6|PB2
A/duck/Korea/HD1/2017|2017-11-17|A/H5N6|PB2
A/chicken/Korea/H203/2018|2018-01-26|A/H5N6|PB2
A/spoonbill/Taiwan/DB645/2017|2017-12-01|A/H5N6|PB2
A/mandarin duck/Korea/H119-4/2017|2017-12-28|A/H5N6|PB2
A/duck/Korea/H35/2017|2017-12-10|A/H5N6|PB2
A/Great Black-backed Gull/Netherlands/1/2017|2017-12-18|A/H5N6|PB2
A/domestic duck/Germany-MV/R9869/2016|2016-11-23|A/H5N8|PB2
A/chicken/Germany-MV/R10048/2016|2016-11-27|A/H5N8|PB2
A/wild duck/Poland/82A/2016|2016-11-02|A/H5N8|PB2
A/chicken/Greece/39 2017/2017|2017-02-06|A/H5N6|PB2
A/duck/Bangladesh/31019/2016|2016-10-24|A/H3N2|PB2
A/wild bird/Jiangxi/P419/2016|2016-01-07|A/H6N8|PB2
A/greylag goose/Germany-NI/AR703-L02138/2017|2017-01-25|A/H5N8|PB2
A/mute swan/Hungary/51049/2016|2016-10-19|A/H5N8|PB2
A/black swan/Germany-BW/R1364/2017|2017-02-12|A/H5N8|PB2
A/mute swan/Croatia/70/2016|2016-10-30|A/H5N8|PB2
A/wigeon/Italy/16VIR9616-3/2016|2016-12-29|A/H5N5|PB2
A/common buzzard/Germany-SN/R1117/2017|2017-02-06|A/H5N5|PB2
A/mute swan/Croatia/102/2016|2016-12-27|A/H5N5|PB2
A/environment/Kamchatka/18/2016|2016-10-01|A/H5N5|PB2
A/breeder duck/Korea/Gochang1/2014|2014-01-16|A/H5N8|PB2
A/common pochard/Germany-BY/AR09-18-L02421/2017|2017-12-28|A/H5N6|PB2
A/Duck/Netherlands/17017237-001-005/2017|2017-12-07|A/H5N6|PB2
A/Black-headed Gull/Netherlands/29/2017|2017-12-18|A/H5N6|PB2
A/canada goose/England/AV58 18OPpoolEP1/2018|2018-01-05|A/H5N6|PB2
A/mallard duck/Netherlands/30/2014|2014-07-21|A/H4N6|PB2
A/mallard duck/Netherlands/16/2012|2012-08-17|A/H3N2|PB2
A/spotbill duck/Korea/WA159/2018|2018-02-01|A/H5N6|PB2
A/chicken/Korea/H214/2018|2018-02-04|A/H5N6|PB2
A/Whooper swan/Xinjiang/13/2020|2020-01-17|A/H5N6|PB2
A/Whooper swan/Mongolia/25/2020|2020-04-30|A/H5N6|PB2
A/Guangdong/18SF020/2018|2018-09-29|A/H5N6|PB2
A/duck/Nghe An/5382VTC/2019|2019-11-29|A/H5N6|PB2
A/common gull/Saratov/1676/2018|2018-10-01|A/H5N6|PB2
A/duck/Taiwan/1702004/2017|2017-02-03|A/H5N6|PB2
A/duck/Hyogo/1/2016|2016-11-14|A/H5N6|PB2
A/chicken/Korea/HN1/2016|2016-11-16|A/H5N6|PB2
A/chicken/Nghe An/14VTC/2015|2015-01-28|A/H5N6|PB2
A/Whooper Swan/Hunan/4/2016|2016-01-27|A/H5N6|PB2
A/muscovy duck/Vietnam/HU7-17/2017|2017-08-01|A/H5N6|PB2
A/Environment/Guangxi/42586/2015|2015-04-02|A/H5N6|PB2
A/gyrfalcon/Washington/41088-6/2014|2014-12-08|A/H5N8|PB2
A/mallard/Korea/W452/2014|2014-02-05|A/H5N8|PB2
A/duck/England/36254/14|2014-11-14|A/H5N8|PB2
A/muscovy duck/Vietnam/HU2-26/2014|2014-12-05|A/H5N6|PB2
A/chicken/Vietnam/NCVD-15A17/2015|2015-03-13|A/H5N6|PB2
A/Sichuan/26221/2014|2014-04-21|A/H5N6|PB2
2016 Qinghai/Uvs-like
2016-18 Eurasian H5N6/N8
2016-17 European H5N5/N8
2017-18 Eurasian H5N6
0.01

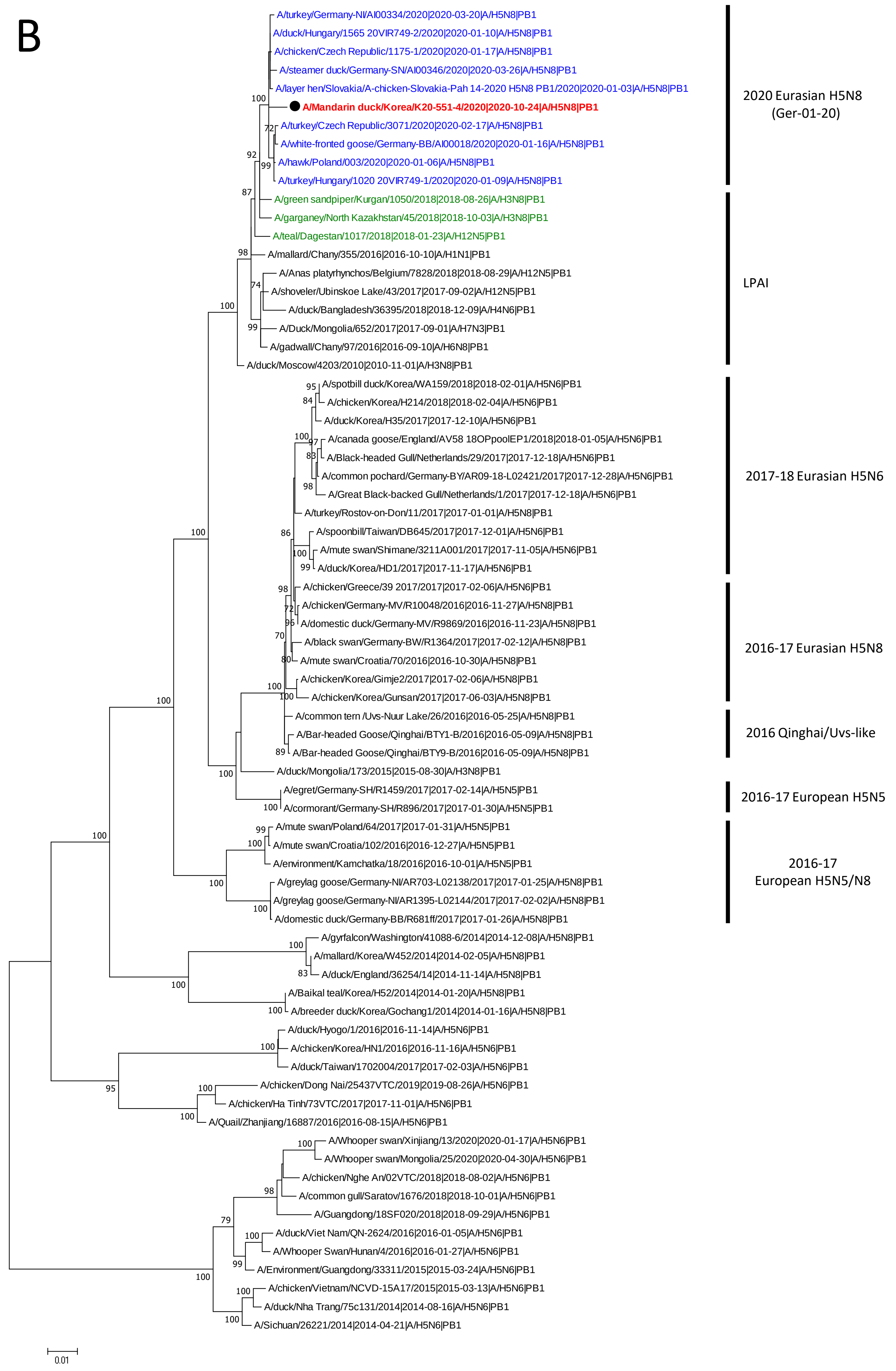
B
A/turkey/Germany-NI/AI00334/2020|2020-03-20|A/H5N8|PB1
A/duck/Hungary/1565 20VIR749-2/2020|2020-01-10|A/H5N8|PB1
A/chicken/Czech Republic/1175-1/2020|2020-01-17|A/H5N8|PB1
A/steamer duck/Germany-SN/AI00346/2020|2020-03-26|A/H5N8|PB1
A/layer hen/Slovakia/A-chicken-Slovakia-Pah 14-2020 H5N8 PB1/2020|2020-01-03|A/H5N8|PB1
A/Mandarin duck/Korea/K20-551-4/2020|2020-10-24|A/H5N8|PB1
A/turkey/Czech Republic/3071/2020|2020-02-17|A/H5N8|PB1
A/white-fronted goose/Germany-BB/AI00018/2020|2020-01-16|A/H5N8|PB1
A/hawk/Poland/003/2020|2020-01-06|A/H5N8|PB1
A/turkey/Hungary/1020 20VIR749-1/2020|2020-01-09|A/H5N8|PB1
A/green sandpiper/Kurgan/1050/2018|2018-08-26|A/H3N8|PB1
A/garganey/North Kazakhstan/45/2018|2018-10-03|A/H3N8|PB1
A/teal/Dagestan/1017/2018|2018-01-23|A/H12N5|PB1
A/mallard/Chany/355/2016|2016-10-10|A/H1N1|PB1
A/Anas platyrhynchos/Belgium/7828/2018|2018-08-29|A/H12N5|PB1
A/shoveler/Ubinskoe Lake/43/2017|2017-09-02|A/H12N5|PB1
A/duck/Bangladesh/36395/2018|2018-12-09|A/H4N6|PB1
A/Duck/Mongolia/652/2017|2017-09-01|A/H7N3|PB1
A/gadwall/Chany/97/2016|2016-09-10|A/H6N8|PB1
A/duck/Moscow/4203/2010|2010-11-01|A/H3N8|PB1
A/spotbill duck/Korea/WA159/2018|2018-02-01|A/H5N6|PB1
A/chicken/Korea/H214/2018|2018-02-04|A/H5N6|PB1
A/duck/Korea/H35/2017|2017-12-10|A/H5N6|PB1
A/canada goose/England/AV58 18OPpoolEP1/2018|2018-01-05|A/H5N6|PB1
A/Black-headed Gull/Netherlands/29/2017|2017-12-18|A/H5N6|PB1
A/common pochard/Germany-BY/AR09-18-L02421/2017|2017-12-28|A/H5N6|PB1
A/Great Black-backed Gull/Netherlands/1/2017|2017-12-18|A/H5N6|PB1
A/turkey/Rostov-on-Don/11/2017|2017-01-01|A/H5N8|PB1
A/spoonbill/Taiwan/DB645/2017|2017-12-01|A/H5N6|PB1
A/mute swan/Shimane/3211A001/2017|2017-11-05|A/H5N6|PB1
A/duck/Korea/HD1/2017|2017-11-17|A/H5N6|PB1
A/chicken/Greece/39 2017/2017|2017-02-06|A/H5N6|PB1
A/chicken/Germany-MV/R10048/2016|2016-11-27|A/H5N8|PB1
A/domestic duck/Germany-MV/R9869/2016|2016-11-23|A/H5N8|PB1
A/black swan/Germany-BW/R1364/2017|2017-02-12|A/H5N8|PB1
A/mute swan/Croatia/70/2016|2016-10-30|A/H5N8|PB1
A/chicken/Korea/Gimje2/2017|2017-02-06|A/H5N8|PB1
A/chicken/Korea/Gunsan/2017|2017-06-03|A/H5N8|PB1
A/common tern /Uvs-Nuur Lake/26/2016|2016-05-25|A/H5N8|PB1
A/Bar-headed Goose/Qinghai/BTY1-B/2016|2016-05-09|A/H5N8|PB1
A/Bar-headed Goose/Qinghai/BTY9-B/2016|2016-05-09|A/H5N8|PB1
A/duck/Mongolia/173/2015|2015-08-30|A/H3N8|PB1
A/egret/Germany-SH/R1459/2017|2017-02-14|A/H5N5|PB1
A/cormorant/Germany-SH/R896/2017|2017-01-30|A/H5N5|PB1
A/mute swan/Poland/64/2017|2017-01-31|A/H5N5|PB1
A/mute swan/Croatia/102/2016|2016-12-27|A/H5N5|PB1
A/environment/Kamchatka/18/2016|2016-10-01|A/H5N5|PB1
A/greylag goose/Germany-NI/AR703-L02138/2017|2017-01-25|A/H5N8|PB1
A/greylag goose/Germany-NI/AR1395-L02144/2017|2017-02-02|A/H5N8|PB1
A/domestic duck/Germany-BB/R681ff/2017|2017-01-26|A/H5N8|PB1
A/gyrfalcon/Washington/41088-6/2014|2014-12-08|A/H5N8|PB1
A/mallard/Korea/W452/2014|2014-02-05|A/H5N8|PB1
A/duck/England/36254/14|2014-11-14|A/H5N8|PB1
A/Baikal teal/Korea/H52/2014|2014-01-20|A/H5N8|PB1
A/breeder duck/Korea/Gochang1/2014|2014-01-16|A/H5N8|PB1
A/duck/Hyogo/1/2016|2016-11-14|A/H5N6|PB1
A/chicken/Korea/HN1/2016|2016-11-16|A/H5N6|PB1
A/duck/Taiwan/1702004/2017|2017-02-03|A/H5N6|PB1
A/chicken/Dong Nai/25437VTC/2019|2019-08-26|A/H5N6|PB1
A/chicken/Ha Tinh/73VTC/2017|2017-11-01|A/H5N6|PB1
A/Quail/Zhanjiang/16887/2016|2016-08-15|A/H5N6|PB1
A/Whooper swan/Xinjiang/13/2020|2020-01-17|A/H5N6|PB1
A/Whooper swan/Mongolia/25/2020|2020-04-30|A/H5N6|PB1
A/chicken/Nghe An/02VTC/2018|2018-08-02|A/H5N6|PB1
A/common gull/Saratov/1676/2018|2018-10-01|A/H5N6|PB1
A/Guangdong/18SF020/2018|2018-09-29|A/H5N6|PB1
A/duck/Viet Nam/QN-2624/2016|2016-01-05|A/H5N6|PB1
A/Whooper Swan/Hunan/4/2016|2016-01-27|A/H5N6|PB1
A/Environment/Guangdong/33311/2015|2015-03-24|A/H5N6|PB1
A/chicken/Vietnam/NCVD-15A17/2015|2015-03-13|A/H5N6|PB1
A/duck/Nha Trang/75c131/2014|2014-08-16|A/H5N6|PB1
A/Sichuan/26221/2014|2014-04-21|A/H5N6|PB1
2020 Eurasian H5N8
(Ger-01-20)
LPAI
2017-18 Eurasian H5N6
2016-17 Eurasian H5N8
2016 Qinghai/Uvs-like
2016-17 European H5N5
2016-17
European H5N5/N8
0.01

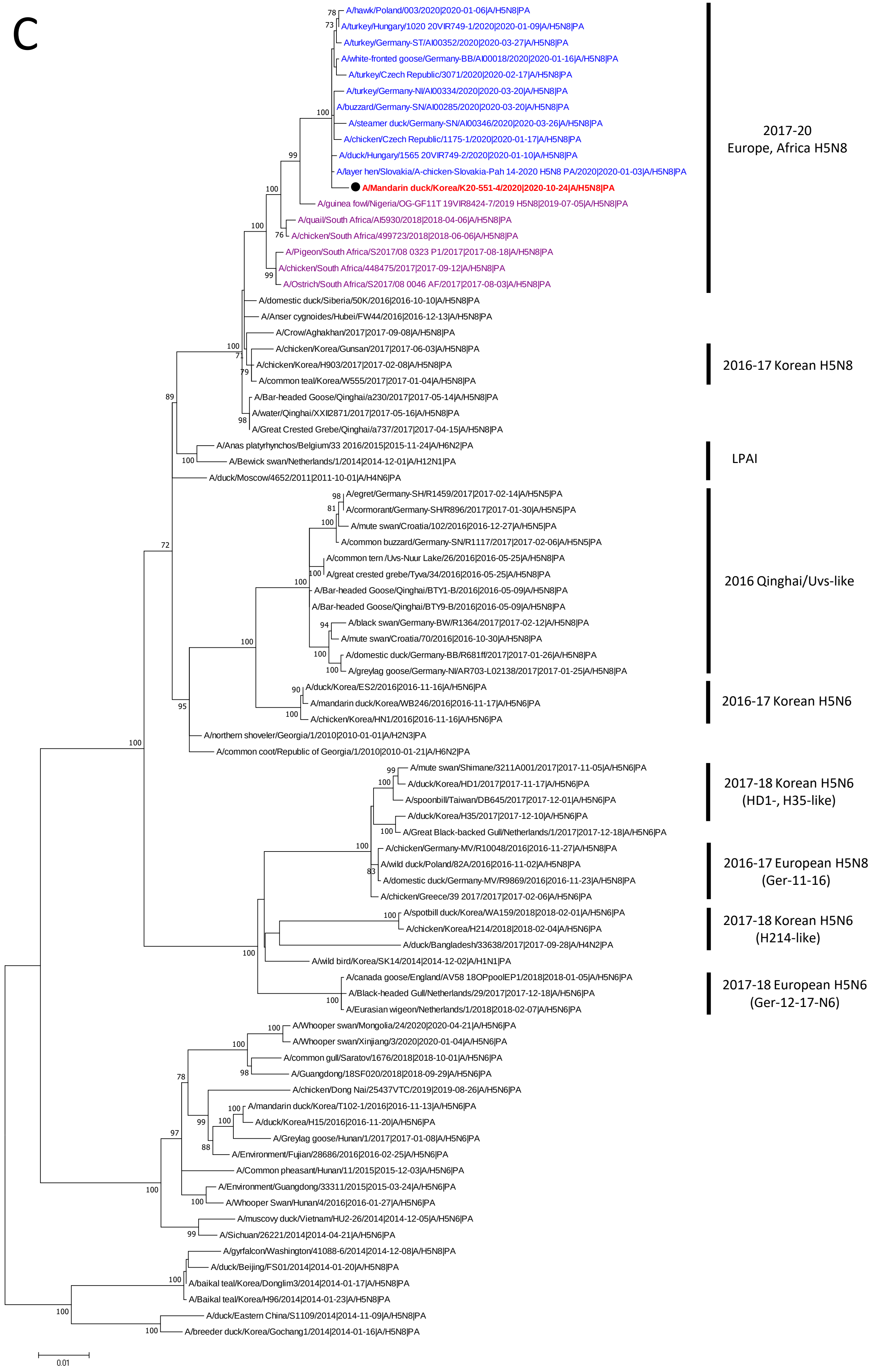
C
A/hawk/Poland/003/2020|2020-01-06|A/H5N8|PA
A/turkey/Hungary/1020 20VIR749-1/2020|2020-01-09|A/H5N8|PA
A/turkey/Germany-ST/AI00352/2020|2020-03-27|A/H5N8|PA
A/white-fronted goose/Germany-BB/AI00018/2020|2020-01-16|A/H5N8|PA
A/turkey/Czech Republic/3071/2020|2020-02-17|A/H5N8|PA
A/turkey/Germany-NI/AI00334/2020|2020-03-20|A/H5N8|PA
A/buzzard/Germany-SN/AI00285/2020|2020-03-20|A/H5N8|PA
A/steamer duck/Germany-SN/AI00346/2020|2020-03-26|A/H5N8|PA
A/chicken/Czech Republic/1175-1/2020|2020-01-17|A/H5N8|PA
A/duck/Hungary/1565 20VIR749-2/2020|2020-01-10|A/H5N8|PA
A/layer hen/Slovakia/A-chicken-Slovakia-Pah 14-2020 H5N8 PA/2020|2020-01-03|A/H5N8|PA
A/Mandarin duck/Korea/K20-551-4/2020|2020-10-24|A/H5N8|PA
A/guinea fowl/Nigeria/OG-GF11T 19VIR8424-7/2019 H5N8|2019-07-05|A/H5N8|PA
A/quail/South Africa/AI5930/2018|2018-04-06|A/H5N8|PA
A/chicken/South Africa/499723/2018|2018-06-06|A/H5N8|PA
A/Pigeon/South Africa/S2017/08 0323 P1/2017|2017-08-18|A/H5N8|PA
A/chicken/South Africa/448475/2017|2017-09-12|A/H5N8|PA
A/Ostrich/South Africa/S2017/08 0046 AF/2017|2017-08-03|A/H5N8|PA
A/domestic duck/Siberia/50K/2016|2016-10-10|A/H5N8|PA
A/Anser cygnoides/Hubei/FW44/2016|2016-12-13|A/H5N8|PA
A/Crow/Aghakhan/2017|2017-09-08|A/H5N8|PA
A/chicken/Korea/Gunsan/2017|2017-06-03|A/H5N8|PA
A/chicken/Korea/H903/2017|2017-02-08|A/H5N8|PA
A/common teal/Korea/W555/2017|2017-01-04|A/H5N8|PA
A/Bar-headed Goose/Qinghai/a230/2017|2017-05-14|A/H5N8|PA
A/water/Qinghai/XXII2871/2017|2017-05-16|A/H5N8|PA
A/Great Crested Grebe/Qinghai/a737/2017|2017-04-15|A/H5N8|PA
A/Anas platyrhynchos/Belgium/33 2016/2015|2015-11-24|A/H6N2|PA
A/Bewick swan/Netherlands/1/2014|2014-12-01|A/H12N1|PA
A/duck/Moscow/4652/2011|2011-10-01|A/H4N6|PA
A/egret/Germany-SH/R1459/2017|2017-02-14|A/H5N5|PA
A/cormorant/Germany-SH/R896/2017|2017-01-30|A/H5N5|PA
A/mute swan/Croatia/102/2016|2016-12-27|A/H5N5|PA
A/common buzzard/Germany-SN/R1117/2017|2017-02-06|A/H5N5|PA
A/common tern /Uvs-Nuur Lake/26/2016|2016-05-25|A/H5N8|PA
A/great crested grebe/Tyva/34/2016|2016-05-25|A/H5N8|PA
A/Bar-headed Goose/Qinghai/BTY1-B/2016|2016-05-09|A/H5N8|PA
A/Bar-headed Goose/Qinghai/BTY9-B/2016|2016-05-09|A/H5N8|PA
A/black swan/Germany-BW/R1364/2017|2017-02-12|A/H5N8|PA
A/mute swan/Croatia/70/2016|2016-10-30|A/H5N8|PA
A/domestic duck/Germany-BB/R681ff/2017|2017-01-26|A/H5N8|PA
A/greylag goose/Germany-NI/AR703-L02138/2017|2017-01-25|A/H5N8|PA
A/duck/Korea/ES2/2016|2016-11-16|A/H5N6|PA
A/mandarin duck/Korea/WB246/2016|2016-11-17|A/H5N6|PA
A/chicken/Korea/HN1/2016|2016-11-16|A/H5N6|PA
A/northern shoveler/Georgia/1/2010|2010-01-01|A/H2N3|PA
A/common coot/Republic of Georgia/1/2010|2010-01-21|A/H6N2|PA
A/mute swan/Shimane/3211A001/2017|2017-11-05|A/H5N6|PA
A/duck/Korea/HD1/2017|2017-11-17|A/H5N6|PA
A/spoonbill/Taiwan/DB645/2017|2017-12-01|A/H5N6|PA
A/duck/Korea/H35/2017|2017-12-10|A/H5N6|PA
A/Great Black-backed Gull/Netherlands/1/2017|2017-12-18|A/H5N6|PA
A/chicken/Germany-MV/R10048/2016|2016-11-27|A/H5N8|PA
A/wild duck/Poland/82A/2016|2016-11-02|A/H5N8|PA
A/domestic duck/Germany-MV/R9869/2016|2016-11-23|A/H5N8|PA
A/chicken/Greece/39 2017/2017|2017-02-06|A/H5N6|PA
A/spotbill duck/Korea/WA159/2018|2018-02-01|A/H5N6|PA
A/chicken/Korea/H214/2018|2018-02-04|A/H5N6|PA
A/duck/Bangladesh/33638/2017|2017-09-28|A/H4N2|PA
A/wild bird/Korea/SK14/2014|2014-12-02|A/H1N1|PA
A/canada goose/England/AV58 18OPpoolEP1/2018|2018-01-05|A/H5N6|PA
A/Black-headed Gull/Netherlands/29/2017|2017-12-18|A/H5N6|PA
A/Eurasian wigeon/Netherlands/1/2018|2018-02-07|A/H5N6|PA
A/Whooper swan/Mongolia/24/2020|2020-04-21|A/H5N6|PA
A/Whooper swan/Xinjiang/3/2020|2020-01-04|A/H5N6|PA
A/common gull/Saratov/1676/2018|2018-10-01|A/H5N6|PA
A/Guangdong/18SF020/2018|2018-09-29|A/H5N6|PA
A/chicken/Dong Nai/25437VTC/2019|2019-08-26|A/H5N6|PA
A/mandarin duck/Korea/T102-1/2016|2016-11-13|A/H5N6|PA
A/duck/Korea/H15/2016|2016-11-20|A/H5N6|PA
A/Greylag goose/Hunan/1/2017|2017-01-08|A/H5N6|PA
A/Environment/Fujian/28686/2016|2016-02-25|A/H5N6|PA
A/Common pheasant/Hunan/11/2015|2015-12-03|A/H5N6|PA
A/Environment/Guangdong/33311/2015|2015-03-24|A/H5N6|PA
A/Whooper Swan/Hunan/4/2016|2016-01-27|A/H5N6|PA
A/muscovy duck/Vietnam/HU2-26/2014|2014-12-05|A/H5N6|PA
A/Sichuan/26221/2014|2014-04-21|A/H5N6|PA
A/gyrfalcon/Washington/41088-6/2014|2014-12-08|A/H5N8|PA
A/duck/Beijing/FS01/2014|2014-01-20|A/H5N8|PA
A/baikal teal/Korea/Donglim3/2014|2014-01-17|A/H5N8|PA
A/Baikal teal/Korea/H96/2014|2014-01-23|A/H5N8|PA
A/duck/Eastern China/S1109/2014|2014-11-09|A/H5N8|PA
A/breeder duck/Korea/Gochang1/2014|2014-01-16|A/H5N8|PA
2017-20 Europe, Africa H5N8
2016-17 Korean H5N8
LPAI
2016 Qinghai/Uvs-like
2016-17 Korean H5N6
2017-18 Korean H5N6 (HD1-, H35-like)
2016-17 European H5N8 (Ger-11-16)
2017-18 Korean H5N6 (H214-like)
2017-18 European H5N6 (Ger-12-17-N6)
0.01

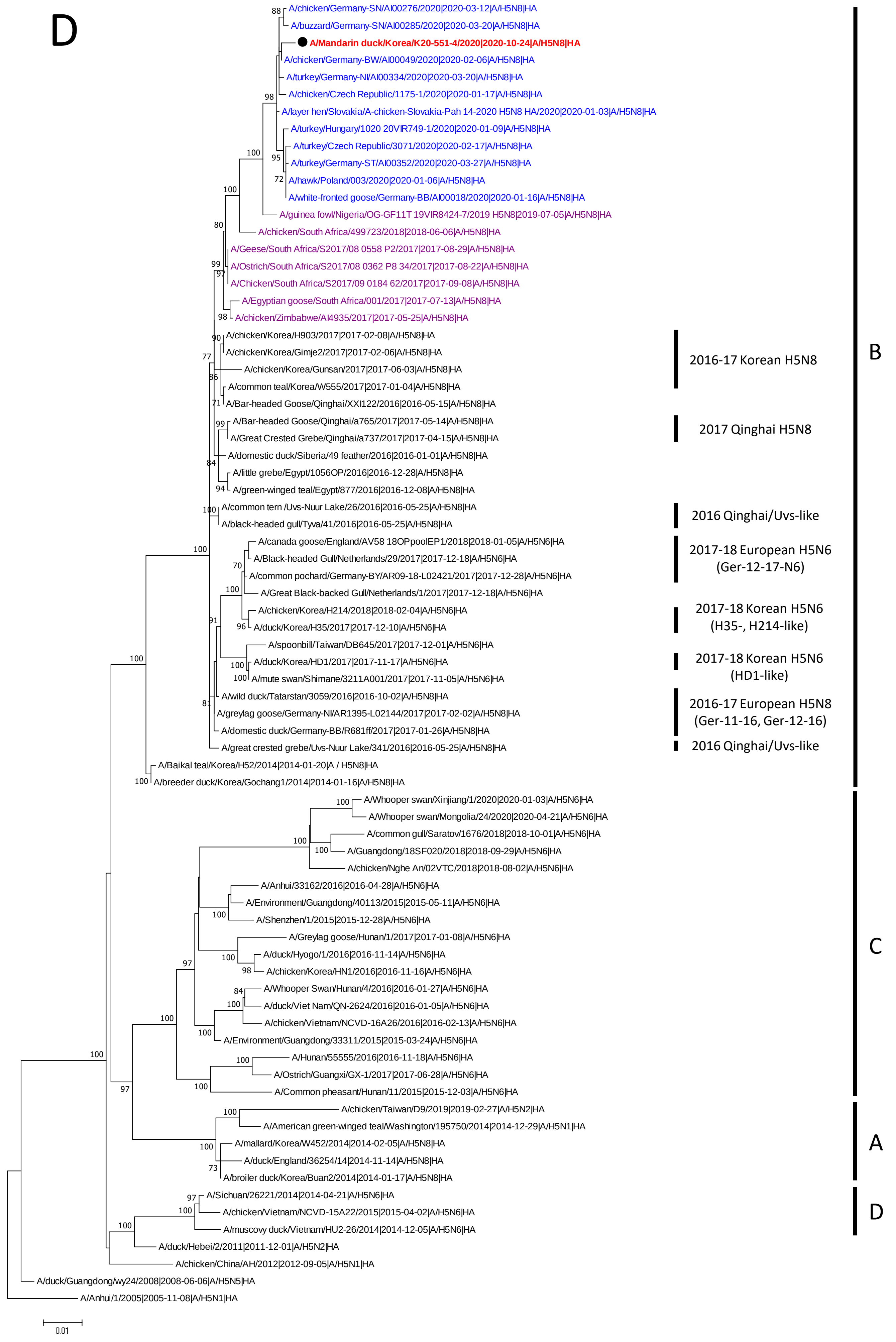
D
A/chicken/Germany-SN/AI00276/2020|2020-03-12|A/H5N8|HA
A/buzzard/Germany-SN/AI00285/2020|2020-03-20|A/H5N8|HA
A/Mandarin duck/Korea/K20-551-4/2020|2020-10-24|A/H5N8|HA
A/chicken/Germany-BW/AI00049/2020|2020-02-06|A/H5N8|HA
A/turkey/Germany-NI/AI00334/2020|2020-03-20|A/H5N8|HA
A/chicken/Czech Republic/1175-1/2020|2020-01-17|A/H5N8|HA
A/layer hen/Slovakia/A-chicken-Slovakia-Pah 14-2020 H5N8 HA/2020|2020-01-03|A/H5N8|HA
A/turkey/Hungary/1020 20VIR749-1/2020|2020-01-09|A/H5N8|HA
A/turkey/Czech Republic/3071/2020|2020-02-17|A/H5N8|HA
A/turkey/Germany-ST/AI00352/2020|2020-03-27|A/H5N8|HA
A/hawk/Poland/003/2020|2020-01-06|A/H5N8|HA
A/white-fronted goose/Germany-BB/AI00018/2020|2020-01-16|A/H5N8|HA
A/guinea fowl/Nigeria/OG-GF11T 19VIR8424-7/2019 H5N8|2019-07-05|A/H5N8|HA
A/chicken/South Africa/499723/2018|2018-06-06|A/H5N8|HA
A/Geese/South Africa/S2017/08 0558 P2/2017|2017-08-29|A/H5N8|HA
A/Ostrich/South Africa/S2017/08 0362 P8 34/2017|2017-08-22|A/H5N8|HA
A/Chicken/South Africa/S2017/09 0184 62/2017|2017-09-08|A/H5N8|HA
A/Egyptian goose/South Africa/001/2017|2017-07-13|A/H5N8|HA
A/chicken/Zimbabwe/AI4935/2017|2017-05-25|A/H5N8|HA
A/chicken/Korea/H903/2017|2017-02-08|A/H5N8|HA
A/chicken/Korea/Gimje2/2017|2017-02-06|A/H5N8|HA
A/chicken/Korea/Gunsan/2017|2017-06-03|A/H5N8|HA
A/common teal/Korea/W555/2017|2017-01-04|A/H5N8|HA
A/Bar-headed Goose/Qinghai/XXI122/2016|2016-05-15|A/H5N8|HA
A/Bar-headed Goose/Qinghai/a765/2017|2017-05-14|A/H5N8|HA
A/Great Crested Grebe/Qinghai/a737/2017|2017-04-15|A/H5N8|HA
A/domestic duck/Siberia/49 feather/2016|2016-01-01|A/H5N8|HA
A/little grebe/Egypt/1056OP/2016|2016-12-28|A/H5N8|HA
A/green-winged teal/Egypt/877/2016|2016-12-08|A/H5N8|HA
A/common tern /Uvs-Nuur Lake/26/2016|2016-05-25|A/H5N8|HA
A/black-headed gull/Tyva/41/2016|2016-05-25|A/H5N8|HA
A/canada goose/England/AV58 18OPpoolEP1/2018|2018-01-05|A/H5N6|HA
A/Black-headed Gull/Netherlands/29/2017|2017-12-18|A/H5N6|HA
A/common pochard/Germany-BY/AR09-18-L02421/2017|2017-12-28|A/H5N6|HA
A/Great Black-backed Gull/Netherlands/1/2017|2017-12-18|A/H5N6|HA
A/chicken/Korea/H214/2018|2018-02-04|A/H5N6|HA
A/duck/Korea/H35/2017|2017-12-10|A/H5N6|HA
A/spoonbill/Taiwan/DB645/2017|2017-12-01|A/H5N6|HA
A/duck/Korea/HD1/2017|2017-11-17|A/H5N6|HA
A/mute swan/Shimane/3211A001/2017|2017-11-05|A/H5N6|HA
A/wild duck/Tatarstan/3059/2016|2016-10-02|A/H5N8|HA
A/greylag goose/Germany-NI/AR1395-L02144/2017|2017-02-02|A/H5N8|HA
A/domestic duck/Germany-BB/R681ff/2017|2017-01-26|A/H5N8|HA
A/great crested grebe/Uvs-Nuur Lake/341/2016|2016-05-25|A/H5N8|HA
A/Baikal teal/Korea/H52/2014|2014-01-20|A / H5N8|HA
A/breeder duck/Korea/Gochang1/2014|2014-01-16|A/H5N8|HA
A/Whooper swan/Xinjiang/1/2020|2020-01-03|A/H5N6|HA
A/Whooper swan/Mongolia/24/2020|2020-04-21|A/H5N6|HA
A/common gull/Saratov/1676/2018|2018-10-01|A/H5N6|HA
A/Guangdong/18SF020/2018|2018-09-29|A/H5N6|HA
A/chicken/Nghe An/02VTC/2018|2018-08-02|A/H5N6|HA
A/Anhui/33162/2016|2016-04-28|A/H5N6|HA
A/Environment/Guangdong/40113/2015|2015-05-11|A/H5N6|HA
A/Shenzhen/1/2015|2015-12-28|A/H5N6|HA
A/Greylag goose/Hunan/1/2017|2017-01-08|A/H5N6|HA
A/duck/Hyogo/1/2016|2016-11-14|A/H5N6|HA
A/chicken/Korea/HN1/2016|2016-11-16|A/H5N6|HA
A/Whooper Swan/Hunan/4/2016|2016-01-27|A/H5N6|HA
A/duck/Viet Nam/QN-2624/2016|2016-01-05|A/H5N6|HA
A/chicken/Vietnam/NCVD-16A26/2016|2016-02-13|A/H5N6|HA
A/Environment/Guangdong/33311/2015|2015-03-24|A/H5N6|HA
A/Hunan/55555/2016|2016-11-18|A/H5N6|HA
A/Ostrich/Guangxi/GX-1/2017|2017-06-28|A/H5N6|HA
A/Common pheasant/Hunan/11/2015|2015-12-03|A/H5N6|HA
A/chicken/Taiwan/D9/2019|2019-02-27|A/H5N2|HA
A/American green-winged teal/Washington/195750/2014|2014-12-29|A/H5N1|HA
A/mallard/Korea/W452/2014|2014-02-05|A/H5N8|HA
A/duck/England/36254/14|2014-11-14|A/H5N8|HA
A/broiler duck/Korea/Buan2/2014|2014-01-17|A/H5N8|HA
A/Sichuan/26221/2014|2014-04-21|A/H5N6|HA
A/chicken/Vietnam/NCVD-15A22/2015|2015-04-02|A/H5N6|HA
A/muscovy duck/Vietnam/HU2-26/2014|2014-12-05|A/H5N6|HA
A/duck/Hebei/2/2011|2011-12-01|A/H5N2|HA
A/chicken/China/AH/2012|2012-09-05|A/H5N1|HA
A/duck/Guangdong/wy24/2008|2008-06-06|A/H5N5|HA
A/Anhui/1/2005|2005-11-08|A/H5N1|HA
0.01
2016-17 Korean H5N8
2017 Qinghai H5N8
2016 Qinghai/Uvs-like
2017-18 European H5N6 (Ger-12-17-N6)
2017-18 Korean H5N6 (H35-, H214-like)
2017-18 Korean H5N6 (HD1-like)
2016-17 European H5N8 (Ger-11-16, Ger-12-16)
2016 Qinghai/Uvs-like
B
C
A
D

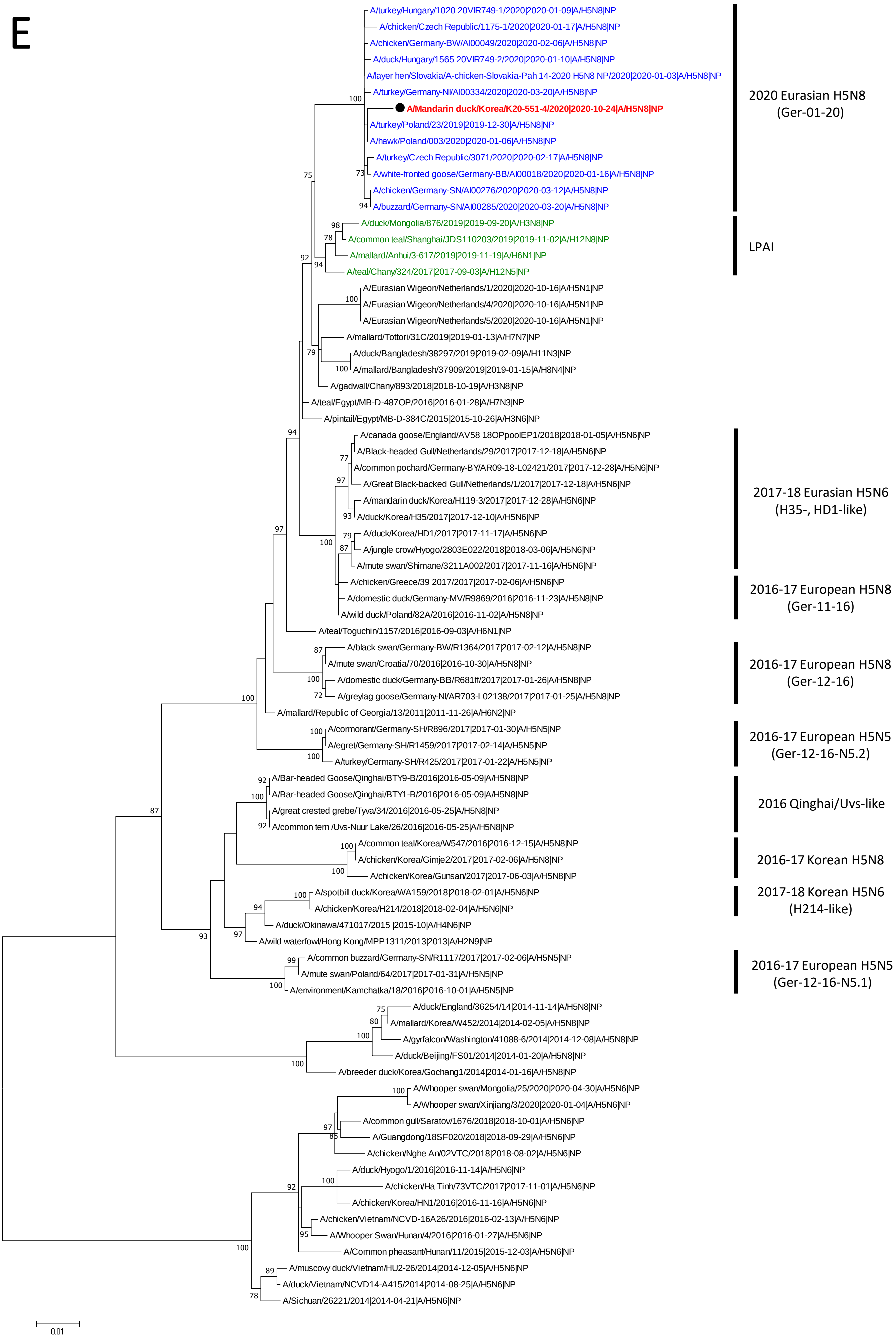
E
A/turkey/Hungary/1020 20VIR749-1/2020|2020-01-09|A/H5N8|NP
A/chicken/Czech Republic/1175-1/2020|2020-01-17|A/H5N8|NP
A/chicken/Germany-BW/AI00049/2020|2020-02-06|A/H5N8|NP
A/duck/Hungary/1565 20VIR749-2/2020|2020-01-10|A/H5N8|NP
A/layer hen/Slovakia/A-chicken-Slovakia-Pah 14-2020 H5N8 NP/2020|2020-01-03|A/H5N8|NP
A/turkey/Germany-NI/AI00334/2020|2020-03-20|A/H5N8|NP
A/Mandarin duck/Korea/K20-551-4/2020|2020-10-24|A/H5N8|NP
A/turkey/Poland/23/2019|2019-12-30|A/H5N8|NP
A/hawk/Poland/003/2020|2020-01-06|A/H5N8|NP
A/turkey/Czech Republic/3071/2020|2020-02-17|A/H5N8|NP
A/white-fronted goose/Germany-BB/AI00018/2020|2020-01-16|A/H5N8|NP
A/chicken/Germany-SN/AI00276/2020|2020-03-12|A/H5N8|NP
A/buzzard/Germany-SN/AI00285/2020|2020-03-20|A/H5N8|NP
2020 Eurasian H5N8
(Ger-01-20)
A/duck/Mongolia/876/2019|2019-09-20|A/H3N8|NP
A/common teal/Shanghai/JDS110203/2019|2019-11-02|A/H12N8|NP
A/mallard/Anhui/3-617/2019|2019-11-19|A/H6N1|NP
A/teal/Chany/324/2017|2017-09-03|A/H12N5|NP
LPAI
A/Eurasian Wigeon/Netherlands/1/2020|2020-10-16|A/H5N1|NP
A/Eurasian Wigeon/Netherlands/4/2020|2020-10-16|A/H5N1|NP
A/Eurasian Wigeon/Netherlands/5/2020|2020-10-16|A/H5N1|NP
A/mallard/Tottori/31C/2019|2019-01-13|A/H7N7|NP
A/duck/Bangladesh/38297/2019|2019-02-09|A/H11N3|NP
A/mallard/Bangladesh/37909/2019|2019-01-15|A/H8N4|NP
A/gadwall/Chany/893/2018|2018-10-19|A/H3N8|NP
A/teal/Egypt/MB-D-487OP/2016|2016-01-28|A/H7N3|NP
A/pintail/Egypt/MB-D-384C/2015|2015-10-26|A/H3N6|NP
A/canada goose/England/AV58 18OPpoolEP1/2018|2018-01-05|A/H5N6|NP
A/Black-headed Gull/Netherlands/29/2017|2017-12-18|A/H5N6|NP
A/common pochard/Germany-BY/AR09-18-L02421/2017|2017-12-28|A/H5N6|NP
A/Great Black-backed Gull/Netherlands/1/2017|2017-12-18|A/H5N6|NP
A/mandarin duck/Korea/H119-3/2017|2017-12-28|A/H5N6|NP
A/duck/Korea/H35/2017|2017-12-10|A/H5N6|NP
A/duck/Korea/HD1/2017|2017-11-17|A/H5N6|NP
A/jungle crow/Hyogo/2803E022/2018|2018-03-06|A/H5N6|NP
A/mute swan/Shimane/3211A002/2017|2017-11-16|A/H5N6|NP
2017-18 Eurasian H5N6
(H35-, HD1-like)
A/chicken/Greece/39 2017/2017|2017-02-06|A/H5N6|NP
A/domestic duck/Germany-MV/R9869/2016|2016-11-23|A/H5N8|NP
A/wild duck/Poland/82A/2016|2016-11-02|A/H5N8|NP
2016-17 European H5N8
(Ger-11-16)
A/teal/Toguchin/1157/2016|2016-09-03|A/H6N1|NP
A/black swan/Germany-BW/R1364/2017|2017-02-12|A/H5N8|NP
A/mute swan/Croatia/70/2016|2016-10-30|A/H5N8|NP
A/domestic duck/Germany-BB/R681ff/2017|2017-01-26|A/H5N8|NP
A/greylag goose/Germany-NI/AR703-L02138/2017|2017-01-25|A/H5N8|NP
2016-17 European H5N8
(Ger-12-16)
A/mallard/Republic of Georgia/13/2011|2011-11-26|A/H6N2|NP
A/cormorant/Germany-SH/R896/2017|2017-01-30|A/H5N5|NP
A/egret/Germany-SH/R1459/2017|2017-02-14|A/H5N5|NP
A/turkey/Germany-SH/R425/2017|2017-01-22|A/H5N5|NP
2016-17 European H5N5
(Ger-12-16-N5.2)
A/Bar-headed Goose/Qinghai/BTY9-B/2016|2016-05-09|A/H5N8|NP
A/Bar-headed Goose/Qinghai/BTY1-B/2016|2016-05-09|A/H5N8|NP
A/great crested grebe/Tyva/34/2016|2016-05-25|A/H5N8|NP
A/common tern /Uvs-Nuur Lake/26/2016|2016-05-25|A/H5N8|NP
2016 Qinghai/Uvs-like
A/common teal/Korea/W547/2016|2016-12-15|A/H5N8|NP
A/chicken/Korea/Gimje2/2017|2017-02-06|A/H5N8|NP
A/chicken/Korea/Gunsan/2017|2017-06-03|A/H5N8|NP
2016-17 Korean H5N8
A/spotbill duck/Korea/WA159/2018|2018-02-01|A/H5N6|NP
A/chicken/Korea/H214/2018|2018-02-04|A/H5N6|NP
2017-18 Korean H5N6
(H214-like)
A/duck/Okinawa/471017/2015 |2015-10|A/H4N6|NP
A/wild waterfowl/Hong Kong/MPP1311/2013|2013|A/H2N9|NP
A/common buzzard/Germany-SN/R1117/2017|2017-02-06|A/H5N5|NP
A/mute swan/Poland/64/2017|2017-01-31|A/H5N5|NP
A/environment/Kamchatka/18/2016|2016-10-01|A/H5N5|NP
2016-17 European H5N5
(Ger-12-16-N5.1)
A/duck/England/36254/14|2014-11-14|A/H5N8|NP
A/mallard/Korea/W452/2014|2014-02-05|A/H5N8|NP
A/gyrfalcon/Washington/41088-6/2014|2014-12-08|A/H5N8|NP
A/duck/Beijing/FS01/2014|2014-01-20|A/H5N8|NP
A/breeder duck/Korea/Gochang1/2014|2014-01-16|A/H5N8|NP
A/Whooper swan/Mongolia/25/2020|2020-04-30|A/H5N6|NP
A/Whooper swan/Xinjiang/3/2020|2020-01-04|A/H5N6|NP
A/common gull/Saratov/1676/2018|2018-10-01|A/H5N6|NP
A/Guangdong/18SF020/2018|2018-09-29|A/H5N6|NP
A/chicken/Nghe An/02VTC/2018|2018-08-02|A/H5N6|NP
A/duck/Hyogo/1/2016|2016-11-14|A/H5N6|NP
A/chicken/Ha Tinh/73VTC/2017|2017-11-01|A/H5N6|NP
A/chicken/Korea/HN1/2016|2016-11-16|A/H5N6|NP
A/chicken/Vietnam/NCVD-16A26/2016|2016-02-13|A/H5N6|NP
A/Whooper Swan/Hunan/4/2016|2016-01-27|A/H5N6|NP
A/Common pheasant/Hunan/11/2015|2015-12-03|A/H5N6|NP
A/muscovy duck/Vietnam/HU2-26/2014|2014-12-05|A/H5N6|NP
A/duck/Vietnam/NCVD14-A415/2014|2014-08-25|A/H5N6|NP
A/Sichuan/26221/2014|2014-04-21|A/H5N6|NP
0.01

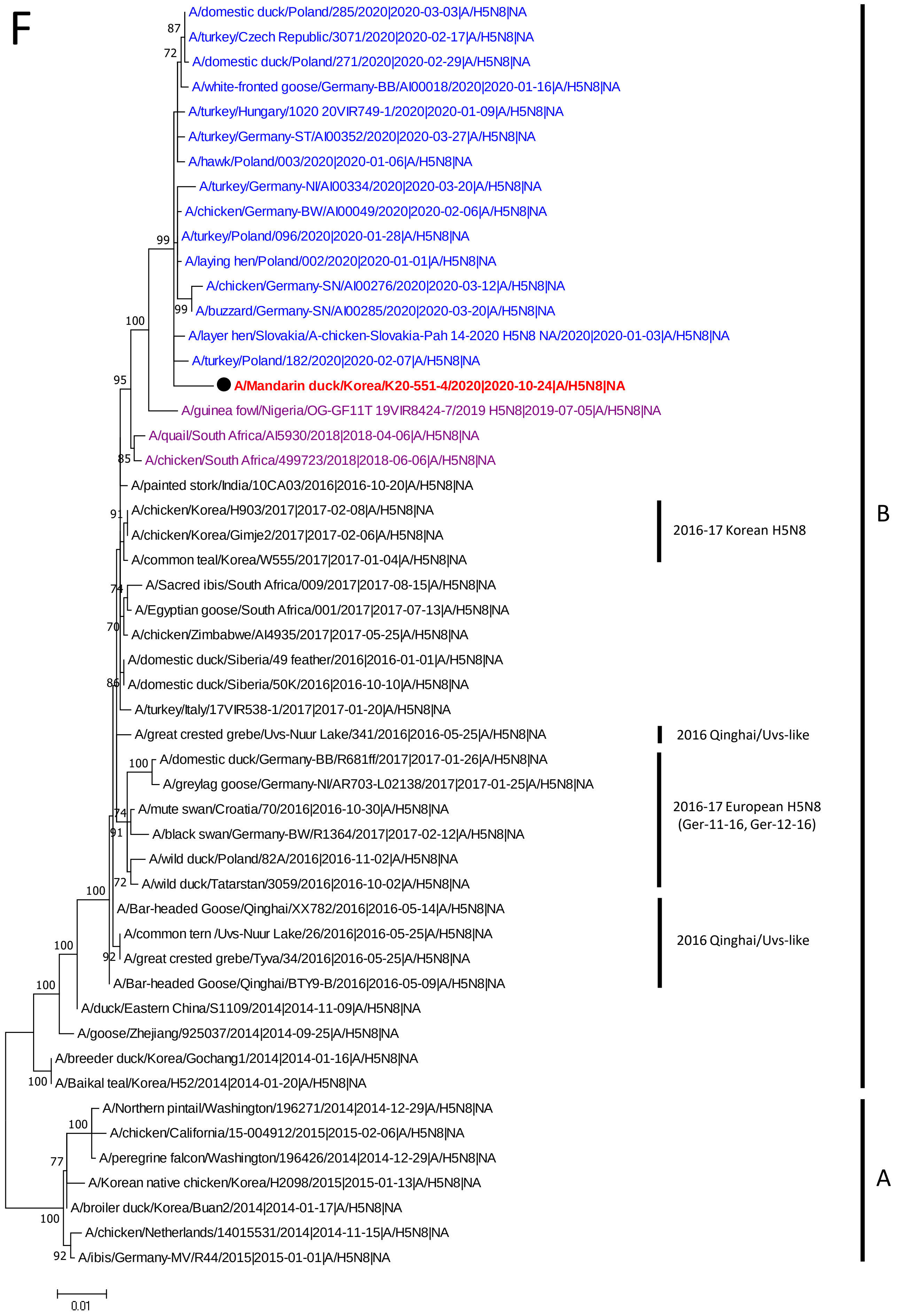

F
A/domestic duck/Poland/285/2020|2020-03-03|A/H5N8|NA
A/turkey/Czech Republic/3071/2020|2020-02-17|A/H5N8|NA
A/domestic duck/Poland/271/2020|2020-02-29|A/H5N8|NA
A/white-fronted goose/Germany-BB/AI00018/2020|2020-01-16|A/H5N8|NA
A/turkey/Hungary/1020 20VIR749-1/2020|2020-01-09|A/H5N8|NA
A/turkey/Germany-ST/AI00352/2020|2020-03-27|A/H5N8|NA
A/hawk/Poland/003/2020|2020-01-06|A/H5N8|NA
A/turkey/Germany-NI/AI00334/2020|2020-03-20|A/H5N8|NA
A/chicken/Germany-BW/AI00049/2020|2020-02-06|A/H5N8|NA
A/turkey/Poland/096/2020|2020-01-28|A/H5N8|NA
A/laying hen/Poland/002/2020|2020-01-01|A/H5N8|NA
A/chicken/Germany-SN/AI00276/2020|2020-03-12|A/H5N8|NA
A/buzzard/Germany-SN/AI00285/2020|2020-03-20|A/H5N8|NA
A/layer hen/Slovakia/A-chicken-Slovakia-Pah 14-2020 H5N8 NA/2020|2020-01-03|A/H5N8|NA
A/turkey/Poland/182/2020|2020-02-07|A/H5N8|NA
A/Mandarin duck/Korea/K20-551-4/2020|2020-10-24|A/H5N8|NA
A/guinea fowl/Nigeria/OG-GF11T 19VIR8424-7/2019 H5N8|2019-07-05|A/H5N8|NA
A/quail/South Africa/AI5930/2018|2018-04-06|A/H5N8|NA
A/chicken/South Africa/499723/2018|2018-06-06|A/H5N8|NA
A/painted stork/India/10CA03/2016|2016-10-20|A/H5N8|NA
A/chicken/Korea/H903/2017|2017-02-08|A/H5N8|NA
A/chicken/Korea/Gimje2/2017|2017-02-06|A/H5N8|NA
A/common teal/Korea/W555/2017|2017-01-04|A/H5N8|NA
A/Sacred ibis/South Africa/009/2017|2017-08-15|A/H5N8|NA
A/Egyptian goose/South Africa/001/2017|2017-07-13|A/H5N8|NA
A/chicken/Zimbabwe/AI4935/2017|2017-05-25|A/H5N8|NA
A/domestic duck/Siberia/49 feather/2016|2016-01-01|A/H5N8|NA
A/domestic duck/Siberia/50K/2016|2016-10-10|A/H5N8|NA
A/turkey/Italy/17VIR538-1/2017|2017-01-20|A/H5N8|NA
A/great crested grebe/Uvs-Nuur Lake/341/2016|2016-05-25|A/H5N8|NA
A/domestic duck/Germany-BB/R681ff/2017|2017-01-26|A/H5N8|NA
A/greylag goose/Germany-NI/AR703-L02138/2017|2017-01-25|A/H5N8|NA
A/mute swan/Croatia/70/2016|2016-10-30|A/H5N8|NA
A/black swan/Germany-BW/R1364/2017|2017-02-12|A/H5N8|NA
A/wild duck/Poland/82A/2016|2016-11-02|A/H5N8|NA
A/wild duck/Tatarstan/3059/2016|2016-10-02|A/H5N8|NA
A/Bar-headed Goose/Qinghai/XX782/2016|2016-05-14|A/H5N8|NA
A/common tern /Uvs-Nuur Lake/26/2016|2016-05-25|A/H5N8|NA
A/great crested grebe/Tyva/34/2016|2016-05-25|A/H5N8|NA
A/Bar-headed Goose/Qinghai/BTY9-B/2016|2016-05-09|A/H5N8|NA
A/duck/Eastern China/S1109/2014|2014-11-09|A/H5N8|NA
A/goose/Zhejiang/925037/2014|2014-09-25|A/H5N8|NA
A/breeder duck/Korea/Gochang1/2014|2014-01-16|A/H5N8|NA
A/Baikal teal/Korea/H52/2014|2014-01-20|A/H5N8|NA
A/Northern pintail/Washington/196271/2014|2014-12-29|A/H5N8|NA
A/chicken/California/15-004912/2015|2015-02-06|A/H5N8|NA
A/peregrine falcon/Washington/196426/2014|2014-12-29|A/H5N8|NA
A/Korean native chicken/Korea/H2098/2015|2015-01-13|A/H5N8|NA
A/broiler duck/Korea/Buan2/2014|2014-01-17|A/H5N8|NA
A/chicken/Netherlands/14015531/2014|2014-11-15|A/H5N8|NA
A/ibis/Germany-MV/R44/2015|2015-01-01|A/H5N8|NA
2016-17 Korean H5N8
2016 Qinghai/Uvs-like
2016-17 European H5N8
(Ger-11-16, Ger-12-16)
2016 Qinghai/Uvs-like
B
A
0.01

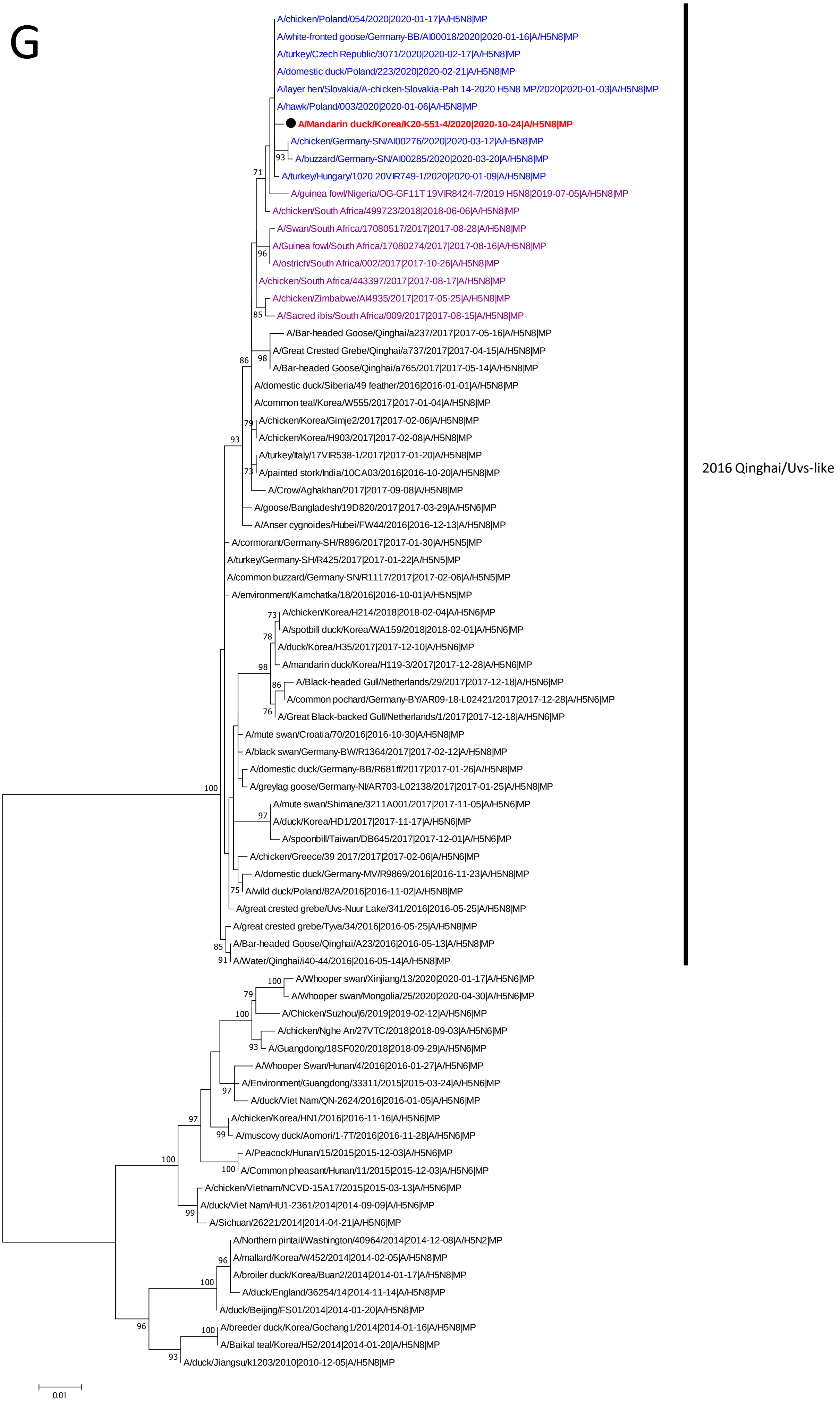
G
A/chicken/Poland/054/2020|2020-01-17|A/H5N8|MP
A/white-fronted goose/Germany-BB/AI00018/2020|2020-01-16|A/H5N8|MP
A/turkey/Czech Republic/3071/2020|2020-02-17|A/H5N8|MP
A/domestic duck/Poland/223/2020|2020-02-21|A/H5N8|MP
A/layer hen/Slovakia/A-chicken-Slovakia-Pah 14-2020 H5N8 MP/2020|2020-01-03|A/H5N8|MP
A/hawk/Poland/003/2020|2020-01-06|A/H5N8|MP
A/Mandarin duck/Korea/K20-551-4/2020|2020-10-24|A/H5N8|MP
A/chicken/Germany-SN/AI00276/2020|2020-03-12|A/H5N8|MP
A/buzzard/Germany-SN/AI00285/2020|2020-03-20|A/H5N8|MP
A/turkey/Hungary/1020 20VIR749-1/2020|2020-01-09|A/H5N8|MP
A/guinea fowl/Nigeria/OG-GF11T 19VIR8424-7/2019 H5N8|2019-07-05|A/H5N8|MP
A/chicken/South Africa/499723/2018|2018-06-06|A/H5N8|MP
A/Swan/South Africa/17080517/2017|2017-08-28|A/H5N8|MP
A/Guinea fowl/South Africa/17080274/2017|2017-08-16|A/H5N8|MP
A/ostrich/South Africa/002/2017|2017-10-26|A/H5N8|MP
A/chicken/South Africa/443397/2017|2017-08-17|A/H5N8|MP
A/chicken/Zimbabwe/AI4935/2017|2017-05-25|A/H5N8|MP
A/Sacred ibis/South Africa/009/2017|2017-08-15|A/H5N8|MP
A/Bar-headed Goose/Qinghai/a237/2017|2017-05-16|A/H5N8|MP
A/Great Crested Grebe/Qinghai/a737/2017|2017-04-15|A/H5N8|MP
A/Bar-headed Goose/Qinghai/a765/2017|2017-05-14|A/H5N8|MP
A/domestic duck/Siberia/49 feather/2016|2016-01-01|A/H5N8|MP
A/common teal/Korea/W555/2017|2017-01-04|A/H5N8|MP
A/chicken/Korea/Gimje2/2017|2017-02-06|A/H5N8|MP
A/chicken/Korea/H903/2017|2017-02-08|A/H5N8|MP
A/turkey/Italy/17VIR538-1/2017|2017-01-20|A/H5N8|MP
A/painted stork/India/10CA03/2016|2016-10-20|A/H5N8|MP
A/Crow/Aghakhan/2017|2017-09-08|A/H5N8|MP
A/goose/Bangladesh/19D820/2017|2017-03-29|A/H5N6|MP
A/Anser cygnoides/Hubei/FW44/2016|2016-12-13|A/H5N8|MP
A/cormorant/Germany-SH/R896/2017|2017-01-30|A/H5N5|MP
A/turkey/Germany-SH/R425/2017|2017-01-22|A/H5N5|MP
A/common buzzard/Germany-SN/R1117/2017|2017-02-06|A/H5N5|MP
A/environment/Kamchatka/18/2016|2016-10-01|A/H5N5|MP
A/chicken/Korea/H214/2018|2018-02-04|A/H5N6|MP
A/spotbill duck/Korea/WA159/2018|2018-02-01|A/H5N6|MP
A/duck/Korea/H35/2017|2017-12-10|A/H5N6|MP
A/mandarin duck/Korea/H119-3/2017|2017-12-28|A/H5N6|MP
A/Black-headed Gull/Netherlands/29/2017|2017-12-18|A/H5N6|MP
A/common pochard/Germany-BY/AR09-18-L02421/2017|2017-12-28|A/H5N6|MP
A/Great Black-backed Gull/Netherlands/1/2017|2017-12-18|A/H5N6|MP
A/mute swan/Croatia/70/2016|2016-10-30|A/H5N8|MP
A/black swan/Germany-BW/R1364/2017|2017-02-12|A/H5N8|MP
A/domestic duck/Germany-BB/R681ff/2017|2017-01-26|A/H5N8|MP
A/greylag goose/Germany-NI/AR703-L02138/2017|2017-01-25|A/H5N8|MP
A/mute swan/Shimane/3211A001/2017|2017-11-05|A/H5N6|MP
A/duck/Korea/HD1/2017|2017-11-17|A/H5N6|MP
A/spoonbill/Taiwan/DB645/2017|2017-12-01|A/H5N6|MP
A/chicken/Greece/39 2017/2017|2017-02-06|A/H5N6|MP
A/domestic duck/Germany-MV/R9869/2016|2016-11-23|A/H5N8|MP
A/wild duck/Poland/82A/2016|2016-11-02|A/H5N8|MP
A/great crested grebe/Uvs-Nuur Lake/341/2016|2016-05-25|A/H5N8|MP
A/great crested grebe/Tyva/34/2016|2016-05-25|A/H5N8|MP
A/Bar-headed Goose/Qinghai/A23/2016|2016-05-13|A/H5N8|MP
A/Water/Qinghai/i40-44/2016|2016-05-14|A/H5N8|MP
2016 Qinghai/Uvs-like
A/Whooper swan/Xinjiang/13/2020|2020-01-17|A/H5N6|MP
A/Whooper swan/Mongolia/25/2020|2020-04-30|A/H5N6|MP
A/Chicken/Suzhou/j6/2019|2019-02-12|A/H5N6|MP
A/chicken/Nghe An/27VTC/2018|2018-09-03|A/H5N6|MP
A/Guangdong/18SF020/2018|2018-09-29|A/H5N6|MP
A/Whooper Swan/Hunan/4/2016|2016-01-27|A/H5N6|MP
A/Environment/Guangdong/33311/2015|2015-03-24|A/H5N6|MP
A/duck/Viet Nam/QN-2624/2016|2016-01-05|A/H5N6|MP
A/chicken/Korea/HN1/2016|2016-11-16|A/H5N6|MP
A/muscovy duck/Aomori/1-7T/2016|2016-11-28|A/H5N6|MP
A/Peacock/Hunan/15/2015|2015-12-03|A/H5N6|MP
A/Common pheasant/Hunan/11/2015|2015-12-03|A/H5N6|MP
A/chicken/Vietnam/NCVD-15A17/2015|2015-03-13|A/H5N6|MP
A/duck/Viet Nam/HU1-2361/2014|2014-09-09|A/H5N6|MP
A/Sichuan/26221/2014|2014-04-21|A/H5N6|MP
A/Northern pintail/Washington/40964/2014|2014-12-08|A/H5N2|MP
A/mallard/Korea/W452/2014|2014-02-05|A/H5N8|MP
A/broiler duck/Korea/Buan2/2014|2014-01-17|A/H5N8|MP
A/duck/England/36254/14|2014-11-14|A/H5N8|MP
A/duck/Beijing/FS01/2014|2014-01-20|A/H5N8|MP
A/breeder duck/Korea/Gochang1/2014|2014-01-16|A/H5N8|MP
A/Baikal teal/Korea/H52/2014|2014-01-20|A/H5N8|MP
A/duck/Jiangsu/k1203/2010|2010-12-05|A/H5N8|MP
0.01

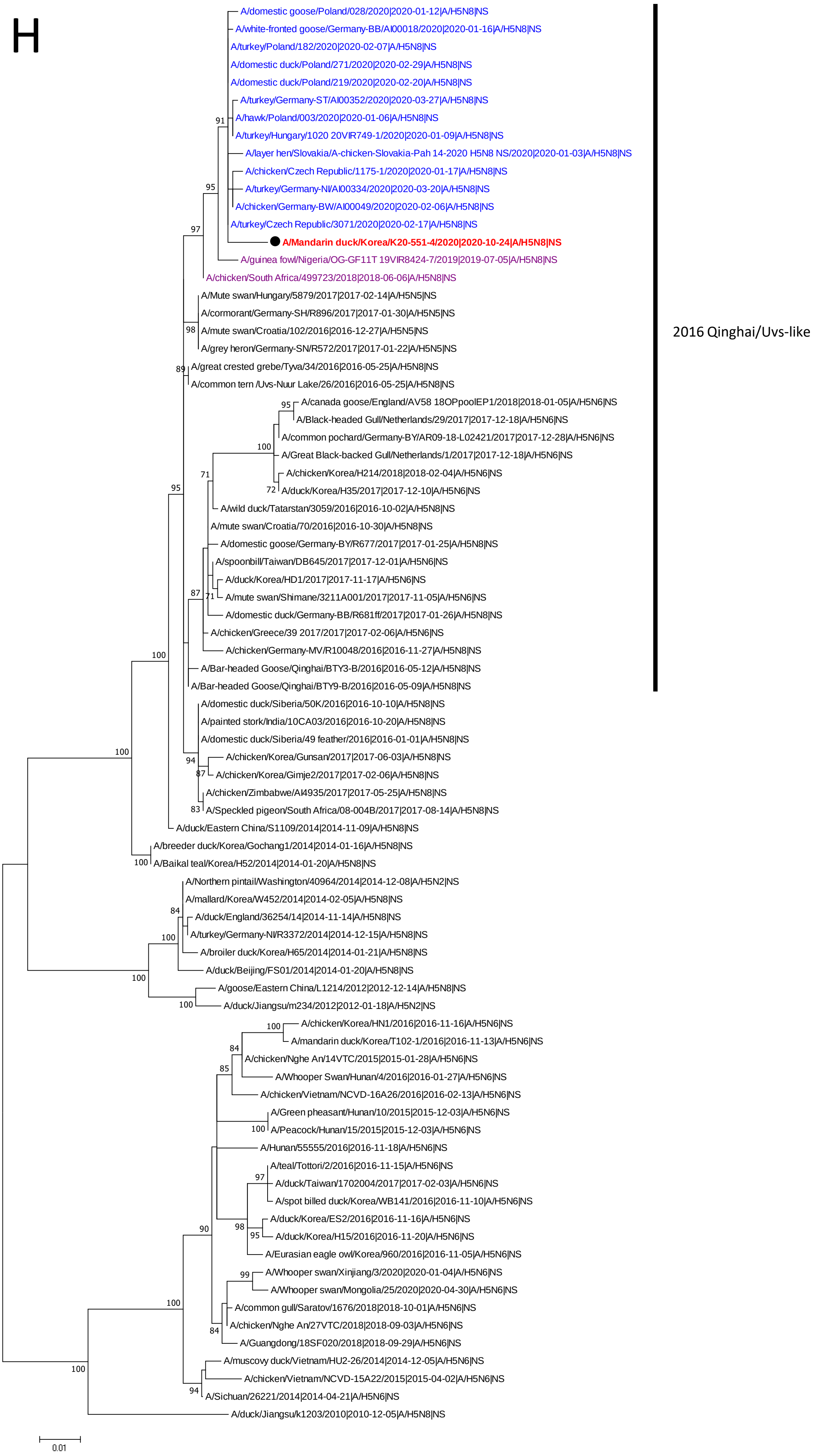
H
A/domestic goose/Poland/028/2020|2020-01-12|A/H5N8|NS
A/white-fronted goose/Germany-BB/AI00018/2020|2020-01-16|A/H5N8|NS
A/turkey/Poland/182/2020|2020-02-07|A/H5N8|NS
A/domestic duck/Poland/271/2020|2020-02-29|A/H5N8|NS
A/domestic duck/Poland/219/2020|2020-02-20|A/H5N8|NS
A/turkey/Germany-ST/AI00352/2020|2020-03-27|A/H5N8|NS
A/hawk/Poland/003/2020|2020-01-06|A/H5N8|NS
A/turkey/Hungary/1020 20VIR749-1/2020|2020-01-09|A/H5N8|NS
A/layer hen/Slovakia/A-chicken-Slovakia-Pah 14-2020 H5N8 NS/2020|2020-01-03|A/H5N8|NS
A/chicken/Czech Republic/1175-1/2020|2020-01-17|A/H5N8|NS
A/turkey/Germany-NI/AI00334/2020|2020-03-20|A/H5N8|NS
A/chicken/Germany-BW/AI00049/2020|2020-02-06|A/H5N8|NS
A/turkey/Czech Republic/3071/2020|2020-02-17|A/H5N8|NS
A/Mandarin duck/Korea/K20-551-4/2020|2020-10-24|A/H5N8|NS
A/guinea fowl/Nigeria/OG-GF11T 19VIR8424-7/2019|2019-07-05|A/H5N8|NS
A/chicken/South Africa/499723/2018|2018-06-06|A/H5N8|NS
A/Mute swan/Hungary/5879/2017|2017-02-14|A/H5N5|NS
A/cormorant/Germany-SH/R896/2017|2017-01-30|A/H5N5|NS
A/mute swan/Croatia/102/2016|2016-12-27|A/H5N5|NS
A/grey heron/Germany-SN/R572/2017|2017-01-22|A/H5N5|NS
A/great crested grebe/Tyva/34/2016|2016-05-25|A/H5N8|NS
A/common tern /Uvs-Nuur Lake/26/2016|2016-05-25|A/H5N8|NS
A/canada goose/England/AV58 18OPpoolEP1/2018|2018-01-05|A/H5N6|NS
A/Black-headed Gull/Netherlands/29/2017|2017-12-18|A/H5N6|NS
A/common pochard/Germany-BY/AR09-18-L02421/2017|2017-12-28|A/H5N6|NS
A/Great Black-backed Gull/Netherlands/1/2017|2017-12-18|A/H5N6|NS
A/chicken/Korea/H214/2018|2018-02-04|A/H5N6|NS
A/duck/Korea/H35/2017|2017-12-10|A/H5N6|NS
A/wild duck/Tatarstan/3059/2016|2016-10-02|A/H5N8|NS
A/mute swan/Croatia/70/2016|2016-10-30|A/H5N8|NS
A/domestic goose/Germany-BY/R677/2017|2017-01-25|A/H5N8|NS
A/spoonbill/Taiwan/DB645/2017|2017-12-01|A/H5N6|NS
A/duck/Korea/HD1/2017|2017-11-17|A/H5N6|NS
A/mute swan/Shimane/3211A001/2017|2017-11-05|A/H5N6|NS
A/domestic duck/Germany-BB/R681ff/2017|2017-01-26|A/H5N8|NS
A/chicken/Greece/39 2017/2017|2017-02-06|A/H5N8|NS
A/chicken/Germany-MV/R10048/2016|2016-11-27|A/H5N8|NS
A/Bar-headed Goose/Qinghai/BTY3-B/2016|2016-05-12|A/H5N8|NS
A/Bar-headed Goose/Qinghai/BTY9-B/2016|2016-05-09|A/H5N8|NS
A/domestic duck/Siberia/50K/2016|2016-10-10|A/H5N8|NS
A/painted stork/India/10CA03/2016|2016-10-20|A/H5N8|NS
A/domestic duck/Siberia/49 feather/2016|2016-01-01|A/H5N8|NS
A/chicken/Korea/Gunsan/2017|2017-06-03|A/H5N8|NS
A/chicken/Korea/Gimje2/2017|2017-02-06|A/H5N8|NS
A/chicken/Zimbabwe/AI4935/2017|2017-05-25|A/H5N8|NS
A/Speckled pigeon/South Africa/08-004B/2017|2017-08-14|A/H5N8|NS
A/duck/Eastern China/S1109/2014|2014-11-09|A/H5N8|NS
A/breeder duck/Korea/Gochang1/2014|2014-01-16|A/H5N8|NS
A/Baikal teal/Korea/H52/2014|2014-01-20|A/H5N8|NS
A/Northern pintail/Washington/40964/2014|2014-12-08|A/H5N2|NS
A/mallard/Korea/W452/2014|2014-02-05|A/H5N8|NS
A/duck/England/36254/14|2014-11-14|A/H5N8|NS
A/turkey/Germany-NI/R3372/2014|2014-12-15|A/H5N8|NS
A/broiler duck/Korea/H65/2014|2014-01-21|A/H5N8|NS
A/duck/Beijing/FS01/2014|2014-01-20|A/H5N8|NS
A/goose/Eastern China/L1214/2012|2012-12-14|A/H5N8|NS
A/duck/Jiangsu/m234/2012|2012-01-18|A/H5N2|NS
A/chicken/Korea/HN1/2016|2016-11-16|A/H5N6|NS
A/mandarin duck/Korea/T102-1/2016|2016-11-13|A/H5N6|NS
A/chicken/Nghe An/14VTC/2015|2015-01-28|A/H5N6|NS
A/Whooper Swan/Hunan/4/2016|2016-01-27|A/H5N6|NS
A/chicken/Vietnam/NCVD-16A26/2016|2016-02-13|A/H5N6|NS
A/Green pheasant/Hunan/10/2015|2015-12-03|A/H5N6|NS
A/Peacock/Hunan/15/2015|2015-12-03|A/H5N6|NS
A/Hunan/55555/2016|2016-11-18|A/H5N6|NS
A/teal/Tottori/2/2016|2016-11-15|A/H5N6|NS
A/duck/Taiwan/1702004/2017|2017-02-03|A/H5N6|NS
A/spot billed duck/Korea/WB141/2016|2016-11-10|A/H5N6|NS
A/duck/Korea/ES2/2016|2016-11-16|A/H5N6|NS
A/duck/Korea/H15/2016|2016-11-20|A/H5N6|NS
A/Eurasian eagle owl/Korea/960/2016|2016-11-05|A/H5N6|NS
A/Whooper swan/Xinjiang/3/2020|2020-01-04|A/H5N6|NS
A/Whooper swan/Mongolia/25/2020|2020-04-30|A/H5N6|NS
A/common gull/Saratov/1676/2018|2018-10-01|A/H5N6|NS
A/chicken/Nghe An/27VTC/2018|2018-09-03|A/H5N6|NS
A/Guangdong/18SF020/2018|2018-09-29|A/H5N6|NS
A/muscovy duck/Vietnam/HU2-26/2014|2014-12-05|A/H5N6|NS
A/chicken/Vietnam/NCVD-15A22/2015|2015-04-02|A/H5N6|NS
A/Sichuan/26221/2014|2014-04-21|A/H5N6|NS
A/duck/Jiangsu/k1203/2010|2010-12-05|A/H5N8|NS
2016 Qinghai/Uvs-like
0.01
