# Supplementary material for: Highly Pathogenic Avian Influenza Clade 2.3.4.4b Subtype H5N8 Virus Isolated from Mandarin Duck in South Korea, 2020
Source: Viruses. 2020 Dec 4;12(12):1389. doi: 10.3390/v12121389 (PMC7761861; doi:10.3390/v12121389)
Supplement: Supplementary file 1 [file viruses-12-01389-s001.zip › supplementary/Supplementary Table.pdf]

Supplementary Table S1: Viruses on which this research is based and the submitters. We would like to thank the research groups that contributed sequence data to the Epiflu Database at GISAID and NCBI's Influenza Virus Resources at GenBank.

Isolate ID/Accession No.	Isolate Name	Submitting Laboratory
EPI ISL 405278	A/guinea fowl/Nigeria/OG-GF11T 19VIR8424-7/2019 H5N8	Istituto Zooprofilattico Sperimentale Delle Venezie
EPI ISL 285653	A/Pigeon/South Africa/S2017/08 0323 P1/2017	National Institute for Communicable Diseases
EPI ISL 285650	A/Ostrich/South Africa/S2017/08 0046 AF/2017	National Institute for Communicable Diseases
EPI ISL 285609	A/Geese/South Africa/S2017/08 0558 P2/2017	National Institute for Communicable Diseases
EPI ISL 285602	A/Geese/South Africa/S2017/09 0065 P2/2017	National Institute for Communicable Diseases
EPI ISL 285917	A/Chicken/South Africa/S2017/09 0184 62/2017	National Institute for Communicable Diseases
EPI ISL 285918	A/Ostrich/South Africa/S2017/08 0362 P8 34/2017	National Institute for Communicable Diseases
EPI ISL 285948	A/Swan/South Africa/S2017/08 0517 P1/2017	National Institute for Communicable Diseases
EPI ISL 285610	A/Guineafowl/South Africa/S2017/08 0274 P1/2017	National Institute for Communicable Diseases
EPI ISL 400491	A/Whooper swan/Shanxi/7/2016	Harbin Veterinary Research Institute (CAAS)
EPI ISL 292479	A/Bar-headed Goose/Qinghai/a893/2017	Wuhan Institute of Virology
EPI ISL 404993	A/white-fronted goose/Germany-BB/AI00018/2020	Friedrich-Loeffler-Institut
EPI ISL 418266	A/turkey/Czech Republic/3071/2020	State Veterinary Institute Prague
EPI ISL 402134	A/turkey/Poland/23/2019	National Veterinary Research Institut Poland, PIWet-PIB
EPI ISL 525440	A/chicken/Poland/003/2020	National Veterinary Research Institut Poland, PIWet-PIB
EPI ISL 419220	A/turkey/Hungary/1020 20VIR749-1/2020	Istituto Zooprofilattico Sperimentale Delle Venezie
EPI ISL 419314	A/turkey/Germany-ST/AI00352/2020	Friedrich-Loeffler-Institut
EPI ISL 419239	A/duck/Hungary/1565 20VIR749-2/2020	Istituto Zooprofilattico Sperimentale Delle Venezie
EPI ISL 405391	A/chicken/Czech Republic/1175-1/2020	State Veterinary Institute Prague
EPI ISL 417414	A/buzzard/Germany-SN/AI00285/2020	Friedrich-Loeffler-Institut
EPI ISL 415197	A/chicken/Germany-SN/AI00276/2020	Friedrich-Loeffler-Institut
EPI ISL 417415	A/turkey/Germany-NI/AI00334/2020	Friedrich-Loeffler-Institut
EPI ISL 419312	A/steamer duck/Germany-SN/AI00346/2020	Friedrich-Loeffler-Institut
EPI ISL 410291	A/chicken/Germany-BW/AI00049/2020	Friedrich-Loeffler-Institut
EPI ISL 525463	A/domestic duck/Poland/285/2020	National Veterinary Research Institut Poland, PIWet-PIB
EPI ISL 525465	A/domestic duck/Poland/271/2020	National Veterinary Research Institut Poland, PIWet-PIB
EPI ISL 525447	A/turkey/Poland/096/2020	National Veterinary Research Institut Poland, PIWet-PIB
EPI ISL 525439	A/laying hen/Poland/002/2020	National Veterinary Research Institut Poland, PIWet-PIB
EPI ISL 525448	A/turkey/Poland/182/2020	National Veterinary Research Institut Poland, PIWet-PIB
EPI ISL 525444	A/chicken/Poland/054/2020	National Veterinary Research Institut Poland, PIWet-PIB
EPI ISL 525452	A/domestic duck/Poland/223/2020	National Veterinary Research Institut Poland, PIWet-PIB
EPI ISL 525443	A/domestic goose/Poland/028/2020	National Veterinary Research Institut Poland, PIWet-PIB
EPI ISL 525449	A/domestic duck/Poland/219/2020	National Veterinary Research Institut Poland, PIWet-PIB
EPI ISL 240678	A/domestic duck/Siberia/50K/2016	Research Institute of Experimental and Clinical Medicine
EPI ISL 240677	A/domestic duck/Siberia/103/2016	Research Institute of Experimental and Clinical Medicine
EPI ISL 288439	A/chicken/Korea/Gimje2/2017	Animal and Plant Quarantine Agency (S-2145)
EPI ISL 288438	A/chicken/Korea/Gunsan/2017	Animal and Plant Quarantine Agency (S-2145)
EPI ISL 247721	A/turkey/Rostov-on-Don/11/2017	State Research Center of Virology and Biotechnology (VECTOR)
EPI ISL 254725	A/chicken/Korea/H903/2017	Animal and Plant Quarantine Agency (S-2026)
EPI ISL 237554	A/painted stork/India/10CA03/2016	ICAR-National Institute of High Security Animal Diseases
EPI ISL 255182	A/turkey/Italy/17VIR538-1/2017	Istituto Zooprofilattico Sperimentale Delle Venezie
EPI ISL 241249	A/domestic duck/Germany-MV/R9869/2016	Friedrich-Loeffler-Institut
EPI ISL 249691	A/chicken/Germany-MV/R10048/2016	Friedrich-Loeffler-Institut
EPI ISL 237921	A/wild duck/Poland/82A/2016	National Veterinary Research Institut Poland, PIWet-PIB
EPI ISL 262059	A/greylag goose/Germany-NI/AR703-L02138/2017	Friedrich-Loeffler-Institut
EPI ISL 237730	A/mute swan/Hungary/51049/2016	Danam.Vet.Molbiol
EPI ISL 261687	A/black swan/Germany-BW/R1364/2017	Friedrich-Loeffler-Institut
EPI ISL 238196	A/mute swan/Croatia/70/2016	Croatian Veterinary Institute
EPI ISL 262058	A/greylag goose/Germany-NI/AR1395-L02144/2017	Friedrich-Loeffler-Institut
EPI ISL 247429	A/domestic duck/Germany-BB/R681ff/2017	Friedrich-Loeffler-Institut
EPI ISL 247724	A/wild duck/Tatarstan/3059/2016	State Research Center of Virology and Biotechnology (VECTOR)

EPI ISL 249682	A/domestic goose/Germany-BY/R677/2017	Friedrich-Loeffler-Institut
EPI ISL 243085	A/wigeon/Italy/16VIR9616-3/2016	Istituto Zooprofilattico Sperimentale Delle Venezie
EPI ISL 259074	A/common buzzard/Germany-SN/R1117/2017	Friedrich-Loeffler-Institut
EPI ISL 240101	A/mute swan/Croatia/102/2016	Croatian Veterinary Institute
EPI ISL 256301	A/environment/Kamchatka/18/2016	State Research Center of Virology and Biotechnology (VECTOR)
EPI ISL 259525	A/egret/Germany-SH/R1459/2017	Friedrich-Loeffler-Institut
EPI ISL 260059	A/cormorant/Germany-SH/R896/2017	Friedrich-Loeffler-Institut
EPI ISL 255917	A/mute swan/Poland/64/2017	National Veterinary Research Institut Poland, PIWet-PIB
EPI ISL 243049	A/turkey/Germany-SH/R425/2017	Friedrich-Loeffler-Institut
EPI ISL 260058	A/grey heron/Germany-SN/R572/2017	Friedrich-Loeffler-Institut
EPI ISL 256462	A/Mute swan/Hungary/5879/2017	Danam.Vet.Molbiol
EPI ISL 335457	A/mute swan/Shimane/3211A002/2017	National Institute of Animal Health
EPI ISL 287800	A/spoonbill/Taiwan/DB645/2017	Animal Health Research Institute
EPI ISL 288437	A/duck/Korea/HD1/2017	Animal and Plant Quarantine Agency (S-2145)
EPI ISL 335450	A/chicken/Korea/H214/2018	Animal and Plant Quarantine Agency (S-2158)
EPI ISL 292345	A/duck/Korea/H35/2017	Animal and Plant Quarantine Agency (S-2145)
EPI ISL 335456	A/spotbill duck/Korea/WA159/2018	Animal and Plant Quarantine Agency (S-2158)
EPI ISL 335448	A/chicken/Korea/H203/2018	Animal and Plant Quarantine Agency (S-2158)
EPI ISL 335440	A/mandarin duck/Korea/H119-4/2017	Animal and Plant Quarantine Agency (S-2158)
EPI ISL 335468	A/Jungle crow/Hyogo/2803E024C/2018	National Institute of Animal Health
EPI ISL 289713	A/Great Black-backed Gull/Netherlands/1/2017	Erasmus Medical Center
EPI ISL 288362	A/chicken/Greece/39 2017/2017	Animal and Plant Health Agency (APHA)
EPI ISL 291109	A/common pochard/Germany-BY/AR09-18-L02421/2017	Friedrich-Loeffler-Institut
EPI ISL 287907	A/Duck/Netherlands/17017237-001-005/2017	Wageningen Bioveterinary Research
EPI ISL 289714	A/Black-headed Gull/Netherlands/29/2017	Erasmus Medical Center
EPI ISL 292225	A/canada goose/England/AV58 18OPpoolEP1/2018	Animal and Plant Health Agency (APHA)
EPI ISL 166693	A/duck/Beijing/FS01/2014	Institute of Microbiology, Chinese Academy of Sciences
EPI ISL 603133	A/Eurasian Wigeon/Netherlands/1/2020	Erasmus Medical Center
EPI ISL 603134	A/Eurasian Wigeon/Netherlands/4/2020	Erasmus Medical Center
EPI ISL 603135	A/Eurasian Wigeon/Netherlands/5/2020	Erasmus Medical Center
EPI ISL 408826	A/Korean native chicken/Korea/H2098/2015	Animal and Plant Quarantine Agency (S-2158)
EPI ISL 418181	A/Whooper swan/Xinjiang/13/2020	Harbin Veterinary Research Institute (CAAS)
EPI ISL 418171	A/Whooper swan/Xinjiang/3/2020	Harbin Veterinary Research Institute (CAAS)
EPI ISL 418169	A/Whooper swan/Xinjiang/1/2020	Harbin Veterinary Research Institute (CAAS)
EPI ISL 337274	A/Guangdong/18SF020/2018	WHO Chinese National Influenza Center
EPI ISL 404208	A/duck/Nghe An/5382VTC/2019	State Research Center of Virology and Biotechnology (VECTOR)
EPI ISL 404987	A/chicken/Dong Nai/25437VTC/2019	State Research Center of Virology and Biotechnology (VECTOR)
EPI ISL 389023	A/chicken/Nghe An/02VTC/2018	State Research Center of Virology and Biotechnology (VECTOR)
EPI ISL 353618	A/Chicken/Suzhou/j6/2019	Jiangsu Provincial Center for Disease Control & Prevention
EPI ISL 389145	A/chicken/Nghe An/27VTC/2018	State Research Center of Virology and Biotechnology (VECTOR)
EPI ISL 224580	A/great crested grebe/Uvs-Nuur Lake/341/2016	Research Institute of Experimental and Clinical Medicine
EPI ISL 234058	A/common tern /Uvs-Nuur Lake/26/2016	Research Institute of Experimental and Clinical Medicine
EPI ISL 230820	A/great crested grebe/Tyva/34/2016	WHO National Influenza Centre Russian Federation
EPI ISL 224704	A/Bar-headed Goose/Qinghai/BTY1-B/2016	Wuhan Institute of Virology
EPI ISL 224709	A/Bar-headed Goose/Qinghai/BTY3-B/2016	Wuhan Institute of Virology
EPI ISL 224721	A/Bar-headed Goose/Qinghai/BTY9-B/2016	Wuhan Institute of Virology
EPI ISL 224729	A/Bar-headed Goose/Qinghai/BTY13-B/2016	Wuhan Institute of Virology
EPI ISL 224751	A/Brown-headed Gull/Qinghai/ZTO6-MU/2016	Wuhan Institute of Virology
EPI ISL 292497	A/Bar-headed Goose/Qinghai/XXI122/2016	Wuhan Institute of Virology
EPI ISL 336925	A/common gull/Saratov/1676/2018	State Research Center of Virology and Biotechnology (VECTOR)
EPI ISL 292192	A/Bar-headed Goose/Qinghai/A23/2016	Wuhan Institute of Virology
EPI ISL 292187	A/Bar-headed Goose/Qinghai/XX782/2016	Wuhan Institute of Virology
EPI ISL 292333	A/Water/Qinghai/i40-44/2016	Wuhan Institute of Virology
EPI ISL 250231	A/domestic duck/Siberia/49 feather/2016	WHO National Influenza Centre Russian Federation
EPI ISL 248634	A/duck/Taiwan/1702004/2017	Animal Health Research Institute
EPI ISL 239351	A/duck/Hyogo/1/2016	National Institute of Infectious Diseases (NIID)

EPI ISL 239261	A/chicken/Korea/HN1/2016(H5N6)	Animal and Plant Quarantine Agency (S-2026)
EPI ISL 278031	A/Quail/Zhanjiang/16887/2016	South China Agricultural University
EPI ISL 239267	A/mandarin duck/Korea/T102-1/2016(H5N6)	Animal and Plant Quarantine Agency (S-2026)
EPI ISL 239263	A/duck/Korea/H15/2016(H5N6)	Animal and Plant Quarantine Agency (S-2026)
EPI ISL 400492	A/Greylag goose/Hunan/1/2017	Harbin Veterinary Research Institute (CAAS)
EPI ISL 283970	A/Ostrich/Guangxi/GX-1/2017 (H5N6)	South China Agricultural University
EPI ISL 240703	A/Hunan/55555/2016	WHO Chinese National Influenza Center
EPI ISL 240533	A/muscovy duck/Aomori/1-7T/2016	National Institute of Animal Health
EPI ISL 239271	A/spot billed duck/Korea/WB141/2016(H5N6)	Animal and Plant Quarantine Agency (S-2026)
EPI ISL 239262	A/duck/Korea/ES2/2016(H5N6)	Animal and Plant Quarantine Agency (S-2026)
EPI ISL 239269	A/mandarin duck/Korea/WB246/2016(H5N6)	Animal and Plant Quarantine Agency (S-2026)
EPI ISL 239266	A/Eurasian eagle owl/Korea/960/2016(H5N6)	Animal and Plant Quarantine Agency (S-2026)
EPI ISL 389082	A/chicken/Nghe An/14VTC/2015	State Research Center of Virology and Biotechnology (VECTOR)
EPI ISL 219800	A/Environment/Guangxi/42586/2015	WHO Chinese National Influenza Center
EPI ISL 400489	A/Whooper Swan/Hunan/4/2016	Harbin Veterinary Research Institute (CAAS)
EPI ISL 163493	A/Sichuan/26221/2014	WHO Chinese National Influenza Center
EPI ISL 389290	A/chicken/Ha Tinh/73VTC/2017	State Research Center of Virology and Biotechnology (VECTOR)
EPI ISL 219813	A/Environment/Guangdong/33311/2015	WHO Chinese National Influenza Center
EPI ISL 333634	A/Environment/Fujian/28686/2016	Fujian Center for Disease Control and Prevention
EPI ISL 400486	A/Common pheasant/Hunan/11/2015	Harbin Veterinary Research Institute (CAAS)
EPI ISL 205314	A/Environment/Shenzhen/1/2015	WHO Chinese National Influenza Center
EPI ISL 219808	A/Environment/Guangdong/40113/2015	WHO Chinese National Influenza Center
EPI ISL 284650	A/Anhui/33162/2016	WHO Chinese National Influenza Center
EPI ISL 333975	A/chicken/Vietnam/NCVD-16A26/2016	Centers for Disease Control and Prevention
EPI ISL 137254	A/duck/Hebei/2/2011	Institute of Microbiology, Chinese Academy of Sciences
EPI ISL 24603	A/Anhui/1/2005	WHO Chinese National Influenza Center
EPI ISL 332682	A/teal/Toguchin/1157/2016	State Research Center of Virology and Biotechnology (VECTOR)
EPI ISL 462612	A/mallard/Anhui/3-617/2019	Harbin Veterinary Research Institute (CAAS)
EPI ISL 331295	A/teal/Chany/324/2017	Research Institute of Experimental and Clinical Medicine
EPI ISL 333615	A/gadwall/Chany/893/2018	State Research Center of Virology and Biotechnology (VECTOR)
EPI ISL 355938	A/green sandpiper/Kurgan/1050/2018	State Research Center of Virology and Biotechnology (VECTOR)
EPI ISL 331307	A/teal/Dagestan/1017/2018	Research Institute of Experimental and Clinical Medicine
EPI ISL 250237	A/mallard/Chany/355/2016	WHO National Influenza Centre Russian Federation
EPI ISL 331306	A/shoveler/Ubinskoe Lake/43/2017	Research Institute of Experimental and Clinical Medicine
EPI ISL 250238	A/gadwall/Chany/97/2016	WHO National Influenza Centre Russian Federation
EPI ISL 237148	A/duck/Okinawa/471017/2015	National Institute of Animal Health
EPI ISL 333132	A/goose/Bangladesh/19D820/2017	Centers for Disease Control and Prevention
EPI ISL 400550	A/Peacock/Hunan/15/2015	Harbin Veterinary Research Institute (CAAS)
EPI ISL 400485	A/Green pheasant/Hunan/10/2015	Harbin Veterinary Research Institute (CAAS)
EPI ISL 237987	A/gyrfalcon/Washington/41088/6/2014	National Institute of Animal Health
EPI ISL 167904	A/duck/England/36254/14	Animal and Plant Health Agency (APHA)
EPI ISL 292340	A/Bar-headed Goose/Qinghai/a230/2017	Wuhan Institute of Virology
EPI ISL 292476	A/Bar-headed Goose/Qinghai/a237/2017	Wuhan Institute of Virology
EPI ISL 292335	A/water/Qinghai/XXII2871/2017	Wuhan Institute of Virology
EPI ISL 292477	A/Great Crested Grebe/Qinghai/a737/2017	Wuhan Institute of Virology
EPI ISL 292478	A/Bar-headed Goose/Qinghai/a765/2017	Wuhan Institute of Virology
EPI ISL 168075	A/chicken/Netherlands/14015531/2014	Wageningen Bioveterinary Research
EPI ISL 172815	A/ibis/Germany-MV/R44/2015	Friedrich-Loeffler-Institut
EPI ISL 169351	A/turkey/Germany-NI/R3372/2014	Friedrich-Loeffler-Institut
MT872354-872361	A/Whooper swan/Mongolia/24/2020	Konkuk University
MT872362-872369	A/Whooper swan/Mongolia/25/2020	Konkuk University
MT027083-027090	A/layer hen/Slovakia/A-chicken-Slovakia-Pah 14-2020/2020	Veterinary Institue Zvolen
MT126633-126637	A/garganey/North Kazakhstan/45/2018	Research Institute for Biological Safety Problems (RIBSP)
